# Machine learning and ontology in eCoaching for personalized activity level monitoring and recommendation generation

**DOI:** 10.1038/s41598-022-24118-4

**Published:** 2022-11-18

**Authors:** Ayan Chatterjee, Nibedita Pahari, Andreas Prinz, Michael Riegler

**Affiliations:** 1 Department of Information and Communication Technology, Centre for e-Health, University of Agder, Jon Lilletuns Vei 9, Grimstad, Norway; 2 Department of Holistic Systems, Simula Metropolitan Center for Digital Engineering (SimulaMet), Oslo, Norway; 3 Department of Software Engineering, Tietoevry Norway AS, Oslo, Norway

**Keywords:** Health care, Mathematics and computing

## Abstract

Leading a sedentary lifestyle may cause numerous health problems. Therefore, passive lifestyle changes should be given priority to avoid severe long-term damage. Automatic health coaching system may help people manage a healthy lifestyle with continuous health state monitoring and personalized recommendation generation with machine learning (ML). This study proposes a semantic ontology model to annotate the ML-prediction outcomes and personal preferences to conceptualize personalized recommendation generation with a hybrid approach. We use a transfer learning approach to improve ML model training and its performance, and an incremental learning approach to handle daily growing data and fit them into the ML models. Furthermore, we propose a personalized activity recommendation algorithm for a healthy lifestyle by combining transfer learning, incremental learning, the proposed semantic ontology model, and personal preference data. For the overall experiment, we use public and private activity datasets collected from healthy adults (n = 33 for public datasets; n = 16 for private datasets). The standard ML algorithms have been used to investigate the possibility of classifying daily physical activity levels into the following activity classes: sedentary (0), low active (1), active (2), highly active (3), and rigorous active (4). The daily step count, low physical activity, medium physical activity, and vigorous physical activity serve as input for the classification models. We first use publicly available Fitbit datasets to build the initial classification models. Subsequently, we re-use the pre-trained ML classifiers on the private MOX2-5 dataset using transfer learning. We test several standard algorithms and select the best-performing model with optimized configuration for our use case by empirical testing. We find that DecisionTreeClassifier with a criterion "entropy” outperforms other ML classifiers with a mean accuracy score of 97.50% (F1 = 97.00, precision = 97.00, recall = 98.00, MCC = 96.78) and 96.10% (F1 = 96.00, precision = 96.00, recall = 96.00, MCC = 96.10) in Fitbit and MOX2-5 datasets, respectively. Using transfer learning, the DecisionTreeClassifier with a criterion "entropy" outperforms other classifiers with a mean accuracy score of 97.99% (F1 = 98.00, precision = 98.00, recall = 98.00, MCC = 96.79). Therefore, the transfer learning approach improves the machine learning model performance by ≈ 1.98% for defined datasets and settings on MOX2-5 datasets. The Hermit reasoner outperforms other reasoners with an average reasoning time of 1.1–2.1 s, under defined settings in our proposed ontology model. Our proposed algorithm for personalized recommendations conceptualizes a direction to combine the classification results and personal preferences in an ontology for activity eCoaching. The proposed method of combining machine learning technology with semantic rules is an invaluable asset in personalized recommendation generation. Moreover, the semantic rules in the knowledge base and SPARQL (SPARQL Protocol and RDF Query Language) query processing in the query engine helps to understand the logic behind the personalized recommendation generation.

## Introduction

According to the World Health Organization (WHO)^1^—an unhealthy lifestyle practice can increase the causes of death worldwide and can double the risk of lifestyle diseases, such as diabetes type II, cardiovascular disease, obesity, elevated blood pressure, cancer, osteoporosis, depression, lipid disorders, and anxiety. Lifestyle diseases are the foremost cause of death worldwide^2,3^. It has been an economic burden to an individual, household, employer, and government and leads to financial and productivity risks for economically poor and rich countries^1–5^.

The idea of activity coaching may improve a personalized healthy lifestyle and physical activity level during the workdays and weekends to reduce sedentary time. The coaching procedure can be “in-person” or “technology-driven” (via telematic means)^6^. In-person coaching with manual activity tracking and feedback generation is time-consuming and monotonous. An automatic coach (eCoach) can generate intuitive and personalized recommendations based on the insight from activity sensor data (as collected with wearable Bluetooth-enabled activity devices, such as Fitbit, MOX2-5) to reach daily, weekly, or monthly goals. Therefore, eHealth monitoring has gained popularity to convey Information and Communication Technology (ICT)-based remote and timely recommendations to the eCoach participants. In this study, we have conceptualized the design of a novel activity eCoaching concept for personalized recommendation generation as a case study that can collect activity data from the participants with wearable activity sensors, process those data with ML models to calculate individual activity levels, integrate personal preference data and ML outcomes in an ontology, and generate personalized recommendations to reach personal activity goals (e.g., daily, weekly, or monthly based on preferences).

### Motivation

Behavior and health are strongly connected. Combining routine activities and nutritious habits can head toward a healthy behavior or lifestyle^7^. Reduction of sedentary time with increased physical activity involves motivation and self-management. Tudor-Locke et al.^8^ and Matthews et al.^9^ showed that people’s activity level differs between weekends and weekdays; on weekdays and Saturdays, people stay active irrespective of gender. Gardner et al.^10^ stated that self-monitoring, problem-solving, and reforming the social or physical environment are the most promising strategies for behavior change besides recommending environmental restructuring, persuasion, and education to improve self-regulation skills. Intervention design to increase physical activity levels and reduce sedentary time varies significantly in content and usefulness (e.g., studies that focus on sports training and behavioral methods show conflicting results, while interventions that reduce sedentary time seem more promising)^10–15^. The use of active video games appears to be efficient in increasing physical activity, but the research results on their suitability to reach recommended levels are inconsistent^16,17^. Web-based or app-based interventions to improve physical activity and reduce sedentary behavior may be effective. The multi-component intervention seems to be more effective than the independent application intervention. However, the optimal number and combination of application functions and the degree of participant exposure need to be confirmed^18,19^. Mobile applications used to improve young people's physical activity should include customized and personalized feedback and provide guidance^20^. Only a few available mHealth apps have been reviewed, and the data is of poor quality^20^. Improving the quality of evidence includes supporting pre-release application performance monitoring, designing experiments, and conducting better reviews through rigorous bias risk assessments^20^. If there is not enough evidence to support it, the practicality of digital interventions and applications will be in its infancy for some time^20^. Workplaces or offices are often used for health promotion interventions. Recent preliminary evidence of workplace interventions to reduce sitting posture at work suggests that alternative workstations (e.g., sit-stand desk or treadmill) can reduce sitting posture in the workplace by thirty minutes to two hours. In addition, one review found that interventions that promote stair use, and personalized behavioral interventions increased physical activity. In contrast, another study found that the various interventions had no significant or inconsistent effects^21,22^.

In physical activity recommendation, an activity tracker is maintained for daily step count, metabolic equivalent of tasks, kilocalories, and distance to reduce sedentary behavior. Data are captured over time and analyzed with ML algorithms to give feedback if the personal activity goals can be achieved or not. The decision module recommends changing a person's behavior, daily routine, and activity plan^10^. The tracker gives an objective measurement of activity level and enables self-monitoring. In addition, most modern consumer-based activity trackers already contain a variety of behavior change models or theories^23,24^. A meta-analysis from Qiu et al.^25^ and Stephenson et al.^26^ concluded that using a pedometer has a small but significant effect on reducing sedentary time. Just wearing an activity tracker (even without any form of guidance) can stimulate the passion for performing physical activities to improve the quality of life.

In contrast, studies on workplace interventions using activity trackers have reported conflicting results^27–29^. Several studies use wearable sensor or activity tracker data to develop customized applications to support research (e.g., the social computer game Fish'n'Steps, which links the daily steps of an employee to the growth and activity of individual fish in a virtual fish tank. The more active, the faster the fish will grow and prosper)^30^. Another example is research on the influence of social support groups using pedometers and mobile apps to increase physical activity^31^.

Different research has studied the use of ML algorithms and sensor data to recognize human activities (e.g., identifying daily activities from an accelerometer signal^32^ or accelerometer-based activity detection^33^, quantification of the lifetime circadian pace of physical activity^34^). Only a few studies have investigated the use of actionable, data-driven predictive models (e.g., a survey to create a predictive physical fatigue model based on sensors has determined the relevant characteristics for predicting physical fatigue; however, the model has not been proven to be sufficiently predictive to be applied^35^). Dijkhuis et al.^15^ performed a study at Hanze University on personalized physical activity coaching with an ML approach to improve sedentary lifestyles. They collected activity data (or daily step data) to train ML classifiers to estimate the probability of achieving hourly step goals and feedback generation with a web-based coaching application. Hansel et al.^36^ designed a fully automated web-based coaching program. They used pedometer-based activity or step monitoring in a random group of Type 2 diabetes and abdominal obesity patients to increase their physical activity. Pessemier et al.^37^ used raw accelerometer data for activity recognition in participants, accepted personal preferences for physical activity recommendation planning, and generated personalized recommendations with a tag-based recommender and rule-based filter. Oliveira et al.^38^ performed activity monitoring with a Fitbit flex activity sensor on a group of random trials. They accomplished a statistical analysis to discover the efficacy of a multimodal physical activity intervention with supervised exercises, health coaching, and activity monitoring on physical activity levels of patients suffering from chronic, nonspecific low back pain. Their study showed that physical activity performs a vital role in managing chronic low back pain.

Semantic rule-based recommendation generation has opened a new direction in eCoaching. Chatterjee et al.^39^ focused on creating a meaningful, context-specific ontology to model non-intuitive, raw, and unstructured observations of health data (e.g., sensors, interviews, and questionnaires) using semantic metadata to create a compact logical abstraction for rule-based health risk assessment for an eCoach paradigm. Villalonga et al.^40^ conceptualized an ontology-based automated reasoning model for generating tailored motivational messages for activity coaching considering behavioral aspects.

Improvement of physical activity in combination with wearable activity sensors and digital activity trackers, eCoach features can be promising and motivating to its participants. An intelligent eCoach system can generate automatic, meaningful, evidence-based, and tailored lifestyle recommendations to attain personal lifestyle goals. The application of machine learning to eCoaching is new, and an electronic search on the PubMed database with a search string: *((ecoach OR e-coach) AND (activity monitoring) AND (Healthy lifestyle or lifestyle) AND (activity or physical activity or exercise) AND (Sensor or activity sensor or activity tracker) AND (recommendation or recommendation generation) AND (data driven or data-driven or classification or prediction or regression or forecasting or rule-based or rule based or ruleset or knowledge base or knowledge-based or hybrid))* has produced no publications. Different activity monitoring and lifestyle coaching mobile applications are available online; however, they lack appropriate design and development guidelines.

### Novelty

The *state-of-the-art* of this study is to conceptualize tailored recommendation generation using ML technology and semantic rules for the management of personalized activity goals. A goal type can be either short-term (e.g., weekly) or long-term (e.g., monthly). The accomplishment of short-term goals attainment may help in achieving the long-term goals.

Limited research has been conducted on sensor data using ML technology (e.g., transfer learning, classification, incremental learning), combining the predictive analysis result and personal preference data with semantic rules for a hybrid recommendation generation. The semantic rules used in this study have shown how to enhance understandability in recommendation generation! The feasibility analysis of ML classifiers and the incremental learning techniques in physical activity recognition have been substantiated to design an ML pipeline. However, this study demonstrates one step ahead by applying them in real-time activity coaching to improve the self-monitoring of actual participants with goal management abilities. This study uses standard ML classification algorithms on the processed sensor data rather than raw signal data for activity level classification. To illustrate the pertinency of the study, we describe a theoretical concept to apply the existing and standard ML classifiers with a semantic ontology for personalized recommendations.

Recommendation technology has a broad application domain. We have considered studies that are only related to lifestyle recommendations, either personal or group-level. Recommendations can be rule-based, data-driven, or hybrid. A qualitative comparison between our study and the related studies has been made in Table 1 based on the following parameters: hybrid recommendations (data-driven and rule-based), transfer learning, incremental learning, observation with activity sensors, and preference settings for tailored recommendation generation. A high-level description of the parameters has been captured in “Appendix A.1”. The study conducted by Pessemier et al. is more focused on community-level activity recommendations, while our research has focused on personal activity coaching with personalized recommendation generation.

**Table 1:** A qualitative comparison between our study and the related lifestyle recommendation studies.

Study	Hybrid recommendation? (Data-driven and rule-based)	Transfer learning	Incremental learning for real-time data processing	Real-time observation with activity sensor	Preference settings for personalized recommendation generation
*Our work*	*Yes*	*Yes*	*Yes*	*Yes*	*Yes*
^15^	Data-driven	No	No	Yes	No
^25^	No	No	No	Yes	No
^26^	No	No	No	Yes	No
^32^	Data-driven	No	No	Yes	No
^33^	Data-driven	No	No	Yes	No
^34^	Data-driven	No	No	Yes	No
^35^	No	No	No	Yes	No
^36^	No	No	No	Yes	No
^37^	Yes	No	No	Yes	Yes
^38^	No	No	No	Yes	No
^39^	Rule-based	No	No	No	No
^40^	Rule-based	No	No	No	No

### Aim of the study

This theoretical study uses ML technology with semantic rules to generate personalized lifestyle recommendations to motivate eCoach participants to accomplish their activity goals. The research questions for this study are:

- *How to use standard ML classifiers with semantic rules in activity coaching for personalized and understandable recommendation generation?*
- *Can transfer learning be useful with an incremental learning approach in low-volume sensor datasets?*

## eCoach prototype system design

**Figure 1:**
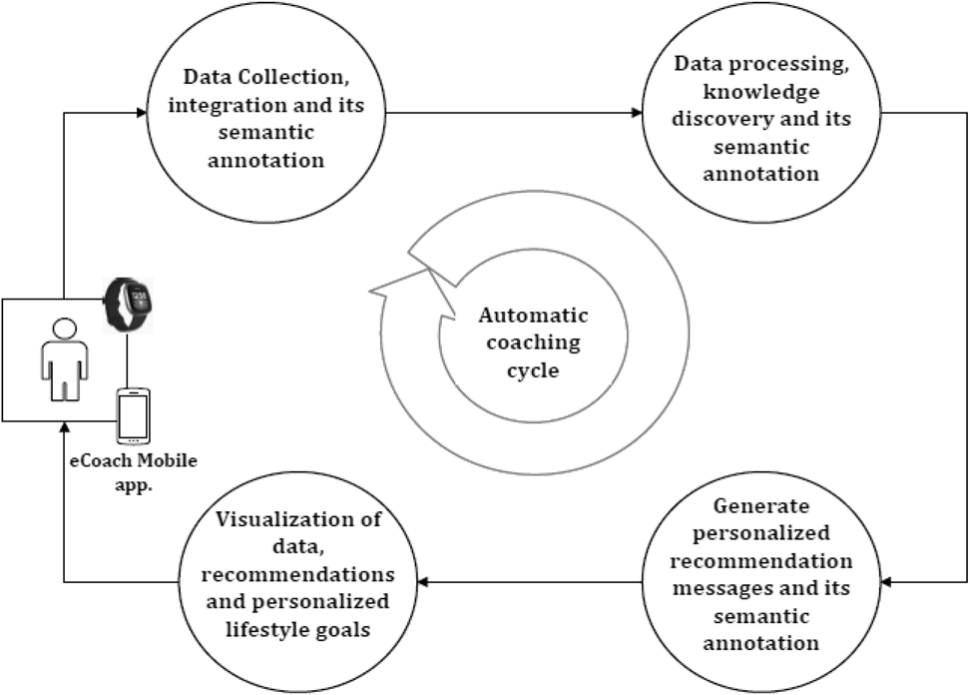
The data flow in our eCoach prototype system.

An eCoach system is a set of computerized components that constitutes an artificial entity that can observe, reason about, learn from and predict a user’s behaviors, in context and over time, and that engages proactively in an ongoing collaborative conversation with the user to aid planning and promote effective goal striving using persuasive techniques^20^. Our eCoach prototype system (see Fig. 1) comprises of the following four modules—data collection and integration, data processing, recommendation generation, and recommendation delivery.

The *data collection and integration module* plan to collect personal preference data through questionnaires and activity data from wearable activity sensors. The *data processing module* analyzes daily activity data and predicts daily activity level with ML models (see Table 2). The personal preference data consists of goal settings (e.g., daily, weekly, or monthly), target goal (e.g., medium active or vigorous active), target score, mode of interaction or recommendation delivery (e.g., text, audio, or graph), and time of recommendation delivery. Individual can modify their preference data, over time. All the collected and predicted personal data are semantically annotated in an ontology model written in the web ontology language (OWL). In the *recommendation generation module*, the ontology helps to generate semantic rule-based recommendation messages with SPARQL query engine. Resource description framework (RDF) uses a triplet structure (subject, predicate, and object) to describe web resources and data exchange. In the created rule base, logical rules comprise of propositional variables with (IMPLIES), (NOT), (AND), and (OR) operations. The rules are of two types: related to activity, and satisfiability. Satisfiability ensures that only one message can be triggered at a time. The recommendation messages are formal and informal (To-Do), and their delivery depends on personal preferences. The *recommendation delivery module* helps to display a reflection of activity in progress with continuous and discrete personal health data, notifications, and recommendation messages in a meaningful way in the eCoach mobile app.

**Table 2:** The rules for “Activity Level” feature creation based on standard guidelines for activity level classification^5^.

Activity level	Rule*	Score
Sedentary	((Steps < 5000) ∧ (VPA*2 + MPA) *7 < 90 ∧ LPA ≥ 0)) ∨ (Steps < 5000)	0
Low active	((Steps > 4999) ∧ (VPA*2 + MPA) *7 ≥ 90 ∧ (VPA*2 + MPA) *7 < 210) ∨ (Steps > 4999 ∧ Steps < 7500)	1
Active	((Steps > 4999) ∧ (VPA*2 + MPA) *7 ≥ 210 ∧ (VPA*2 + MPA) *7 < 300) ∨ (Steps > 7499 ∧ Steps < 10,000)	2
Medium active	((Steps > 4999) ∧ (VPA*2 + MPA) *7 ≥ 300 ∧ (VPA*2 + MPA) *7 < 360)) ∨ (Steps > 9999 ∧ Steps < 12,500)	3
Highly active	((Steps > 4999) ∧ (VPA*2 + MPA) *7 ≥ 360) ∨ (Steps > 12,499)	4

*MPA = 2VPA.

## Proposed work

### Ontology modeling

**Figure 2:**
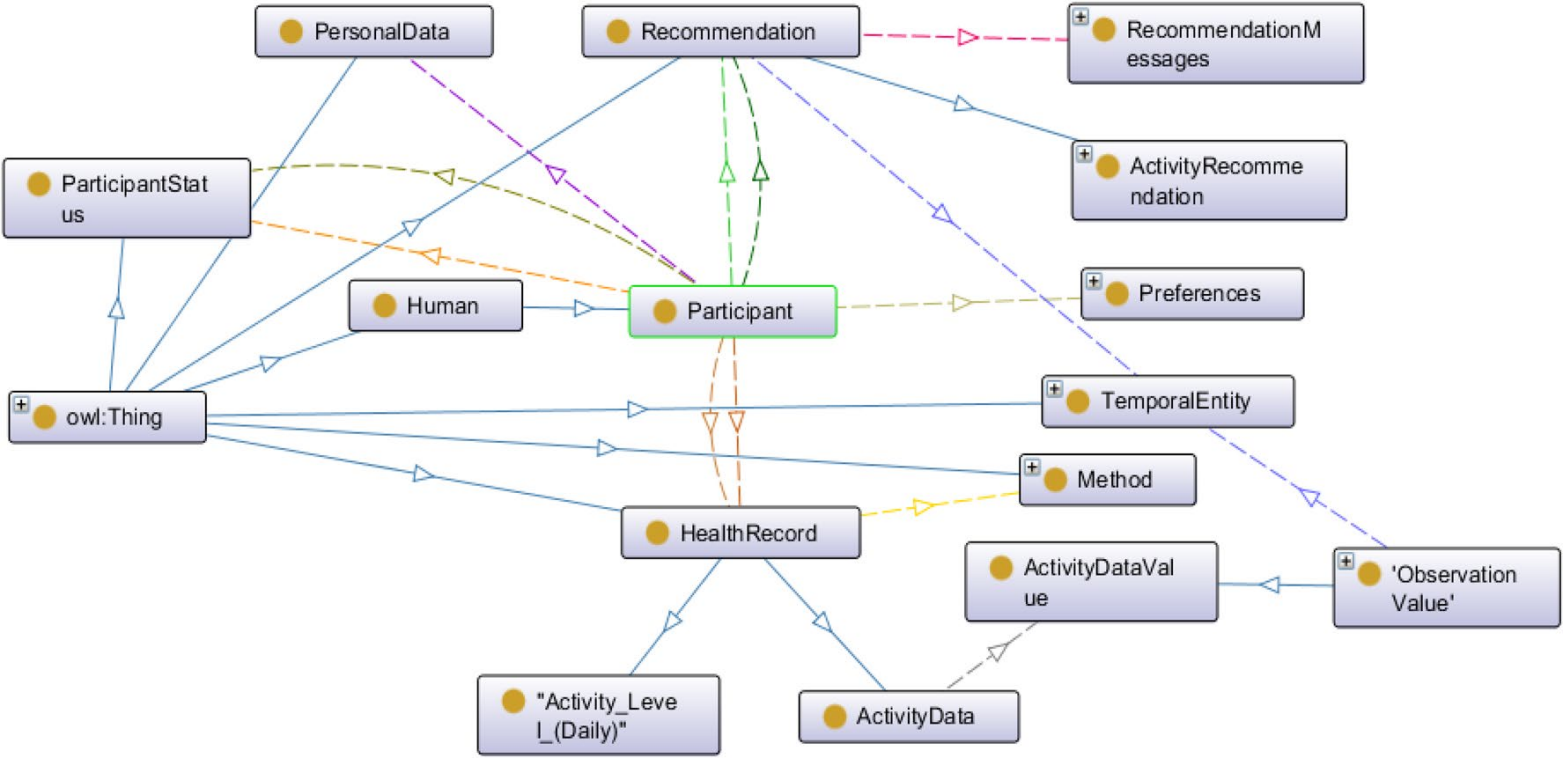
The high-level structure of the proposed ontology.

**Figure 3:**
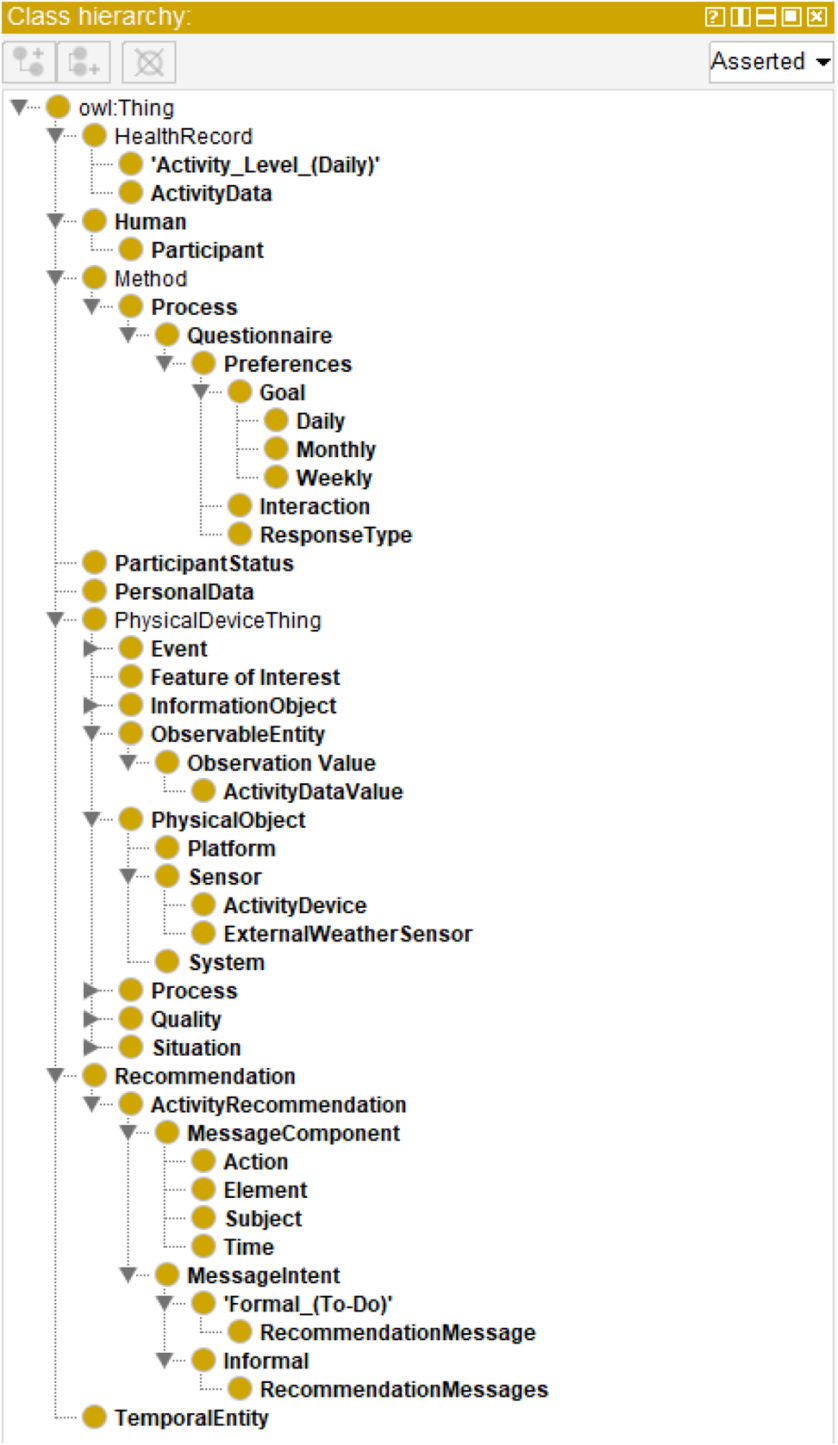
The asserted class hierarchy of our proposed ontology.

The idea of ontology was created thousands of years ago in the philosophical domain. It has the design flexibility and can use existing ontologies to solve real-world modeling and knowledge representation problems. It supports an open-world assumption (OWA) knowledge representation style with the following elements: classes, objects, properties, relationships, and axioms^39,41^. Properties are of two types: ObjectProperties and DataProperties. Each property has a domain-range, restriction rule, restriction filter, and restriction type as Some (existential), Only (Universal), Min (Minimum Cardinality), Exact (Exact Cardinality), and Max (Max Cardinality). An ontology follows a connected, acyclic, and directed tree structure^39,41^. Owl:Thing acts as a super-class in an ontology class hierarchy. Our ontology has been explained in Textbox 1 and its high-level structure has been depicted in Fig. 2 using OntoGraf tool in Protégé. The asserted class hierarchy of the ontology has been depicted in Fig. 3. The objectProperties, domain, range, property type, and cardinality of the ontology are defined in Table 3.

**Table 3:** Key objectProperties, domain, range, and cardinality of the ontology.

Object properties	Domain	Range	Cardinality
hasHealthRecord	Participant	HealthRecord	Some
hasPersonalData	Participant	PersonalData	Some
hasPreferences	Participant	Preferences	Some
hasReceivedRecommendation	Participant	Recommendation	Some
hasStatus	Participant	ParticipantStatus	Some
hasbeenCollectedBy	ActivityData	ActivityDataValue	Some
hasTimeStamp	ActivityDataValue, Questionnaire, Recommendation	TemporalEntity	Some
has Measurement Capability	ActivityDevice	Measurement Capability	Only
hasOutput	ActivityDevice	Sensor Output	Some
observes	ActivityDevice	Property	Only
detects	ActivityDevice	Stimulus	Only
feature of interest	Observation	Feature of Interest	Only
observation result	Observation	Sensor Output	Only
observedBy	Observation	Sensor	Only
is property of	Property	Feature of Interest	Some
hasProperty	Feature of Interest	Property	Some

**Figure 4:**
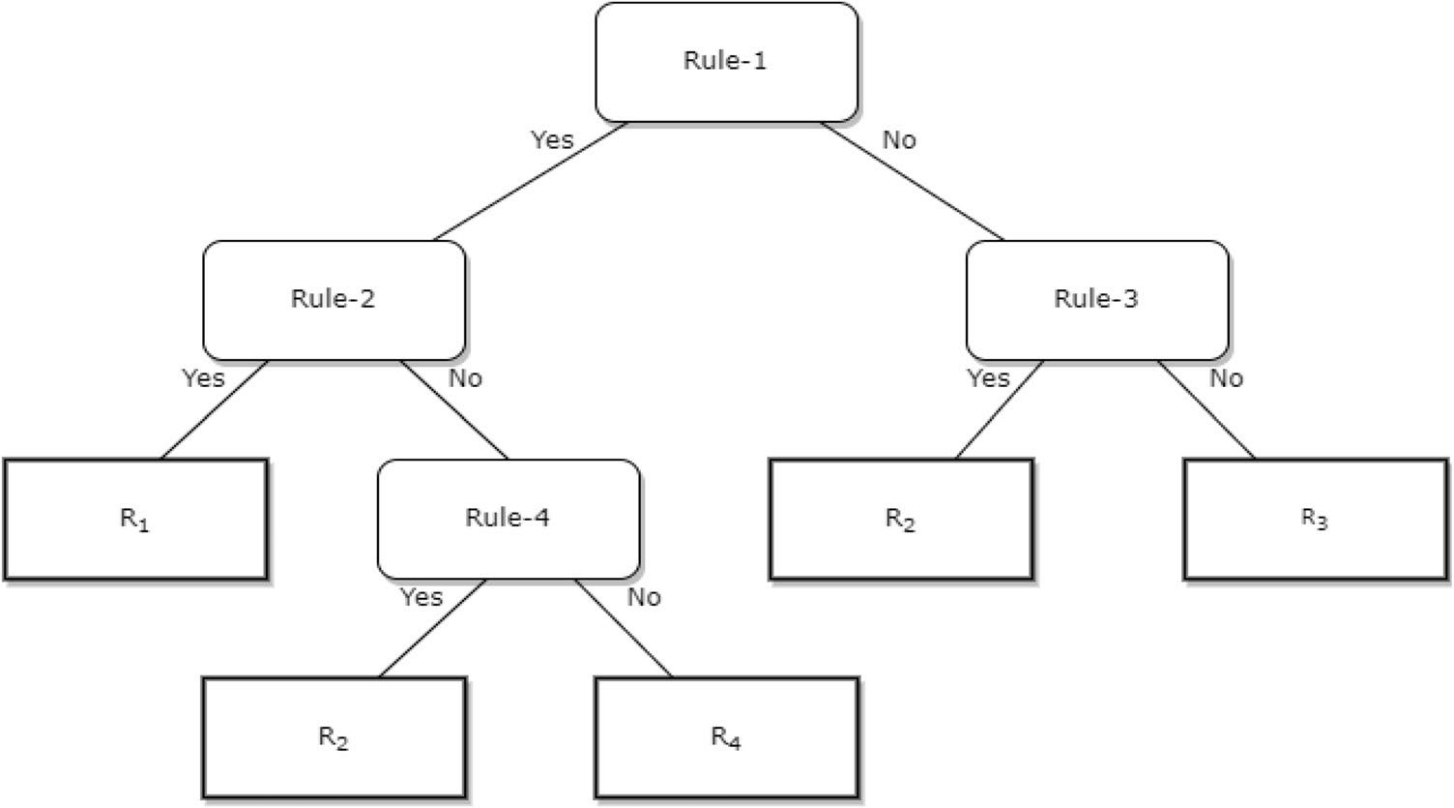
The structure of the recommendation generation binary tree.

The purpose of the ontology is semantic representation of the knowledge (such as activity data, recommendation, and ML prediction outcomes), reasoning, and rule-based decision making with the generalization rules in the induction phase. The proposed ontology follows the following knowledge representation phases: abstraction or lexicon phase (L) for mapping rules, abduction phase (B) for hypothesis generation rule, deduction phase (C) for operator-reductor rule, and induction phase (D) for generalization rule. The resultant recommendation generation tree (T) follows a binary structure, and the syntactic knowledge representation in T helps to address understandability problem in personalized recommendation generation (see Fig. 4).

A set of propositional variables, logics, constants, and operators (such as NOT, AND, OR, IMPLIES, EQUIV, and quantifiers) are linked with Ontology representation and processing. In this study, the recommendation generation aims to maximize weekly individual physical activity level to minimize sedentary time. The maximization problem to stay medium activate for a week ((∑ days (1…0.7))) has been expressed in Textbox 2.

According to the World Health Organization (WHO) guidelines, adults (age:18–64) should do at least 150–300 min (2.5–5 h) of moderate-intensity aerobic exercise (MPA); or at least 75–150 min of high-intensity aerobic exercise (VPA) or perform an equivalent combination of moderate and high-intensity activities within a week to stay active^5^. To determine the weekly score of personal goal achievement, we have summed up the daily activity score (see Table 2). eCoaching aims goal score maximization with constant activity monitoring and recommendation generation. To conceptualize the personalized recommendation generation in our eCoach system, we have considered an example of personal preferences table (see Table 4).

**Table 4:** Personalized preferences for participants.

Preferences	Value
Goal setting	Weekly score
Nature of goal	System defined—Generic [set by the WHO]
Frequency of recommendation delivery	Weekly
Target goal	To stay medium active for the entire week
Target score	21
Mode of recommendation	Text (e.g., push notification on the eCoach app.)
Time of recommendation	8:00 am

Our proposed ontology has integrated daily activity level classification results, personal preferences, and recommendation messages with its content and intent. The activity goals can be system-defined (i.e., generic) or user-defined, as athletes may have different activity goals than ordinary people. For the verification, we have used the ontology for automatic rule-based recommendation generation with SPARQL queries and semantic rulesets maintained in a knowledge base. The ontology has annotated recommendation messages beyond static literal to describe its characteristics, metadata, and content. Additionally, the semantic rules have helped to interpret the logic behind recommendation generation with logical (AND), (OR), and (NOT) operations. “Appendix A.2” describes a set of defined recommendation messages for ontology verification. However, the rules are adaptable. SROIQ description logic has been used as the conventional logic for reasoning (see “Appendix A.3”). For each condition described in “Appendix A.3”, the recommendations will be generated following the *((Rule) IMPLIES (Proposition variable)* → *Recommendation message)* structure. This study has divided six semantic rules into activity level classification (5) and satisfiability (1).

Quantifiable parameters associated with certain participants' activities on the timestamp are obtained from SPARQL queries at preference-based intervals. The rules (1–6) in “Appendix A.3” assign truth values to proposition variables to ensure reliability. We have confirmed that the correct recommendation message will be triggered for a specific context with the ontology reasoner. Therefore, it is important to guarantee that no propositional variable pattern makes the entire rule unsatisfiable. We have established that only one message will be triggered at a time. Here, we have assumed that two “once a day” messages can neither be initiated concurrently nor can there be a model output by the ontology reasoner every time for every possible variable combination. Suppose we put the different variables used in the first five rules in “Appendix A.3” into the propositional variables (see “Appendix A.3”). In that case, it will produce an exponential number of “possible participants”. A typical way to ensure the presence of a model negates all our rules and provides the same. Thus, this formula is truly unsatisfactory. Since two recommendation messages cannot be triggered concurrently to meet the exact requirements, we have added a rule (Rule-6), and the variable used in the proposal starts “once a day”. If (Rule-6) is false, the entire ruleset (considered as significant conjunction) will lead to false, and then there will be no model as output, and we will be able to "debug" our rules if required. If it is set to true, we will have a formal assurance that no matter the true value we put in the knowledge base, two “once a day” messages will not be triggered concurrently. All the rule execution internally follows a binary tree structure where the non-leaf nodes hold the semantic rules (A | A → B) to be executed (see Fig. 4), and the root node has the condition (A). The leaf nodes contain the results (B or recommendations), and the edges hold a decision statement (True or False). In this manner, satisfiability and understandability problems are addressed in this study.

### Personalized activity recommendation generation

For personalized activity recommendation generation, the updating processes for the global (G_C_) and local (L_C_) classifiers are described in Algorithm 1. Models (L_C_) trained with personalized activity data are disjointed with the trained models for other participants for a month as datasets are separate and personal. A global classifier (G_C_) is re-built at the end of every month, combining all individual’s monthly historical activity data. The process is repeated to handle growing data and increase the model's learning, stability, and performance. The process is stopped for an individual if their eCoaching cycle is ended.
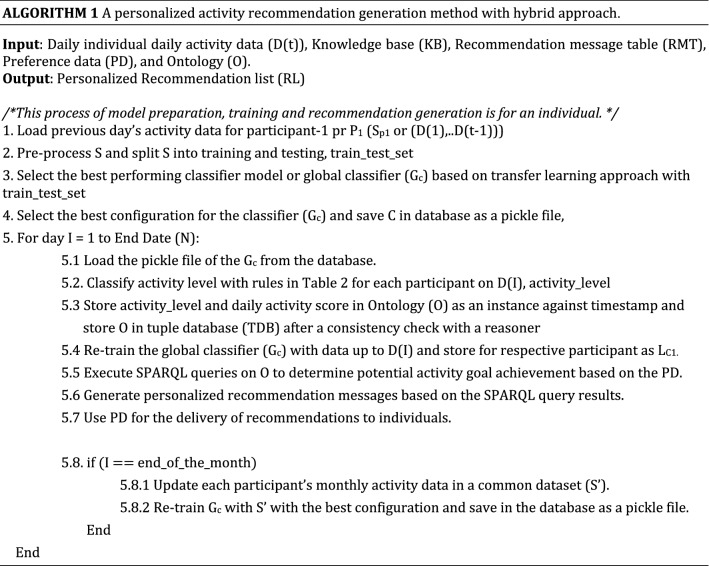

Textbox 1. The Ontology and Knowledge Expression An ontology can be defined as a tuple Ω = {Ć, R}, where Ć is the set of concepts and R is a set of relations^41^.

- L = Levels (O_h_) = Total number of levels in the ontology hierarchy, 0 ≤ n ≤ L, where n ⋲ Z^+^ and n = 0 represents the root node.
- C_n,j_ = a model classifying O at a level n; where, j ⋲ {0, 1, …, |C_n_|}
- |C|= Number of instances classified as class C
- E = Edge (C_n,j_, C_n-1, k_) = edge between node C_n,j_ and its parent node C_n-1, k_

We have used the concept and represented our ontology with four tuples:

O = {C_a_, R, I, A}

- C_a_: {C_a1_, C_a2_, ….., C_an_} represents “n” concepts or classes and each C_ai_ has a set of “j” attributes or properties A_i_ = {a_1_, a_2_,…….., a_j_} provided n, i, j ⋲ Z^+^.
- R: A set of binary relations between the elements of Ca. It holds two subsets –
  - H: Inheritance relationship among concepts
  - S: Semantic relationship between concepts with a domain and range
- I: Represents a knowledge base with set of object instances.
- A: Represents a set of axioms to model O. A includes domain specific constraints to model an Ontology with C_a_, R, and I.

The knowledge (K) in the ontology has been expressed with two tuples:

K = {Onto_ActivityReco_, Rules_ActivityReco_},

- The elements of OntoActivityReco and RulesActivityReco are:
- Onto_ActivityReco_ = {K_L_, K_B_, K_C_, K_D_}
- Rules_ActivityReco_ = {R_L_, R_B_, R_C_, R_D_}

K_L_, K_B_, K_C_, K_D_ are the knowledge bases of the personalized physical activity recommendation’s lexicon or abstraction, abduction, deduction, and induction interfaces. In contrast, R_L_, R_B_, R_C_, R_D_ are set of rules to match with the abstraction, abduction, deduction, and induction interfaces, respectively. K_B_, K_C_, and K_D_ are representations of properties A of concepts (C), data or entities (e.g., activity variables), and they follow a simple representation of A(X|Y) or A(Y|X) based on the relational mapping; where, A: Attributes or properties in O, X, Y: Elements of activity variables. All the rule execution internally follows a binary tree structure where the non-leaf nodes hold the semantic rules (A | A → B) to be executed, the leaf nodes hold the results (B or recommendation messages), and the edges hold a decision statement (True or False). Rulesets help to explain the logic behind a recommendation generation.

Textbox 2. Expression for the activity maximization problem Maximize,

$$\begin{aligned} & \sum {\text{Moderate}}_{{{\text{Activitytime}}}} > {15}0 \\ & \sum {\text{GoalScore}}_{{{\text{daily}}}} \ge {21} \\ \end{aligned}$$

Subject to,

$$\begin{aligned} & {\text{Moderate}}_{{{\text{Activitytime}}}} \ge {21}.{45} \\ & {\text{GoalScore}}_{{{\text{daily}}}} \ge {3} \\ & {\text{C}}_{{\text{V}}} \to {\text{P}} \\ & {\text{P}} \to {\text{R}} \\ & \sum {\text{P}} = {1} \\ & {\text{Moderate}}_{{{\text{Activitytime}}}} = {2}*{\text{Vigorous}}_{{{\text{Activitytime}}}} \\ \end{aligned}$$

where,

- V = A set of activity variables = {V1, V2, ………………., Vn | n ⋲ Z +}
- P = A set of propositional variables = {P1, P2, ………………., Pn | n ⋲ Z +}
- CV = Set of activity variable combinations to create semantic rules in a description logic
- R = A set of recommendations = {R1, R2, ……………….., Rn | RX ⋂ Ry = {ø}, n, x, y ⋲ Z +}
- ∑ P = 1 ensures satisfiability

## Methods

In this study, we have established a theoretical eCoaching concept for personalized activity monitoring, goal management, and lifestyle recommendation generation following the standard guidelines. For the same, we have used established statistical methods and ML models to analyze both public and private activity datasets for adults (age range 18–64). Afterwards, we have shown a direction to combine the result of the classifiers with semantic rules to generate personalized recommendation for automatic activity coaching.

**Figure 5:**
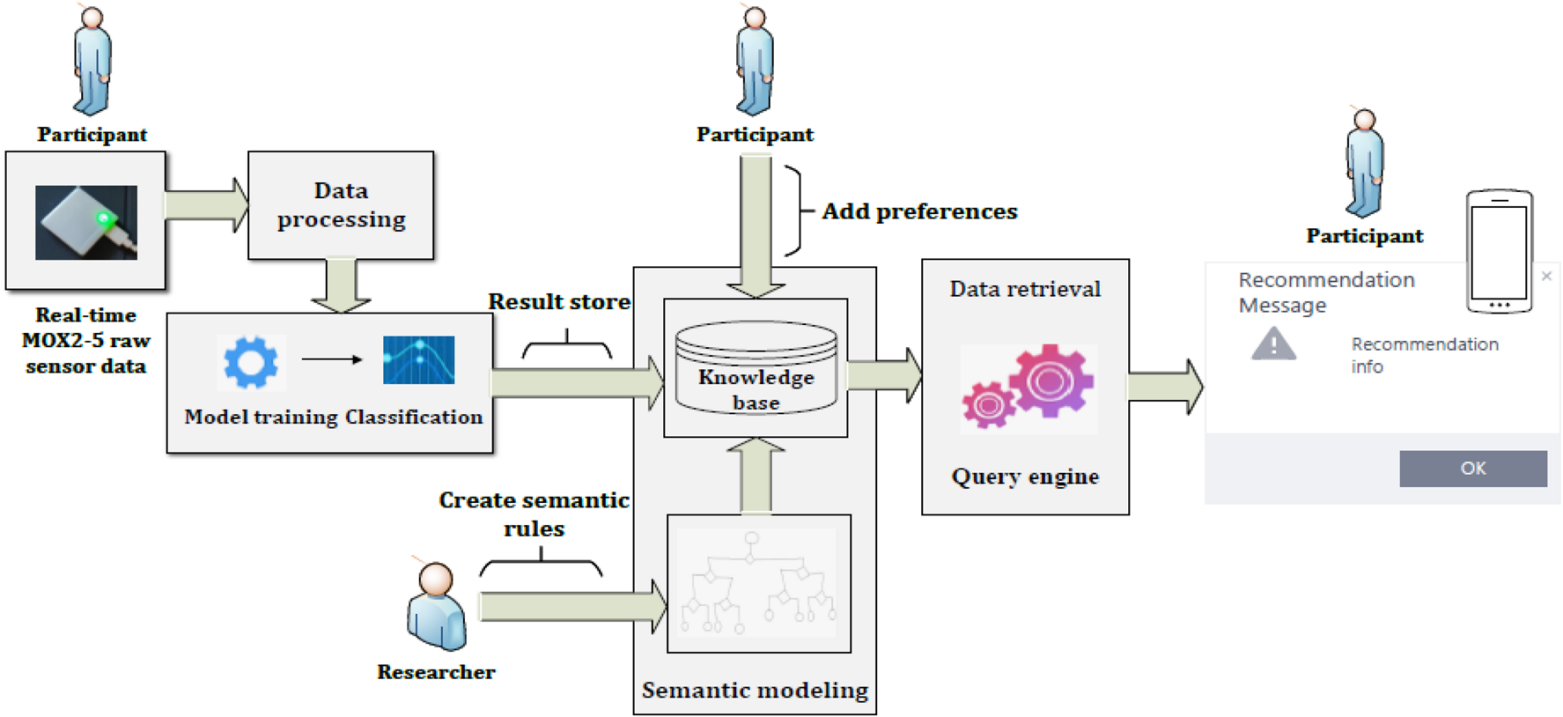
The process of combining classification result with semantic rules and preferences for personalized recommendation generation.

The overall process (see Fig. 5) includes data collection, data pre-processing, semantic presentation of data, statistical analysis, data visualization, feature ranking and selection, ML model training, testing, and evaluation, and semantic rules for personalized recommendation generation. Activity data for elderly, children, athlete, bodybuilder, and pregnant women are beyond the scope of this study as we have not collected data for them. We have followed the Standards for Reporting Implementation (StaRI) for this study (see “Appendix B”). All methods have been carried out following the regulations, and relevant guidelines in the “Ethical approval and consent to participate” section.

### Data collection

#### Public fitbit dataset

We have used an anonymous Fitbit dataset (“dailyActivity_merged”) for adults available via “Zenodo”^42^ for the initial ML model training and testing. The description of the dataset is outlined in “Appendix A.4”. We found two datasets from: mturkfitbit_export_3.12.16-4.11.16 and mturkfitbit_export_4.12.16-5.12.16. Therefore, we have merged two datasets into a single dataset. The dataset has various features related to the activity; however, we have selected the following relevant features—timestamp, total daily steps, LPA minutes, MPA minutes, VPA minutes, and sedentary minutes based on feature analysis to maintain the focus of this study. We have used this public dataset to discover the best-performing classifiers with the defined features in a multiclass classification problem. Then, we applied the best performing model to the real-time datasets using transfer learning and an incremental learning approach for daily activity level classification.

#### Private MOX2-5 dataset

We have collected anonymous activity data from sixteen adults in Sothern-Norway (Agder region) for one month using the MOX2-5 wearable medical-grade (CE approved) activity sensor^43^ following the ethical guidelines and a signed consent. Based on an agreement with “University of Agder, Grimstad, Norway”, NSD—The Norwegian Centre for Research Data AS has assessed that the processing of personal data in this project is in accordance with data protection legislation. The detailed description of the MOX2-5 dataset is summarized in “Appendix A.5”.

After feature analysis the selected features are timestamp, activity intensity (IMA), sedentary seconds, weight-bearing seconds, standing seconds, LPA seconds, MPA seconds, VPA seconds, and steps per minute. The “step” and “IMA” are the most valuable and strong features of the MOX2-5 datasets, as other attributes (except the timestamp) are almost derived (e.g., LPA, MPA, and VPA are derived from IMA as defined in Table 5). IMA has a strong relation with steps. In MOX2-5 sensor, sedentary time refers to the non-activity duration, including leisure time and sleep time. The relation between sedentary and active (LPA/MPA/VPA) can be written as—

1 $$\sum \left( {{\text{sedentary}},\;{\text{active}},\;{\text{weight}} - {\text{bearing}},\,{\text{standing}}} \right) = 60\,{\text{seconds}}$$

**Table 5:** Relation between IMA and activity level classification.

Activity type	Rule
LPA	0 ≤ IMA ≤ 400
MPA	401 ≤ IMA ≤ 800
VPA	IMA ≥ 801

### Data processing and preparation

#### Nature of data and data volume

The selected temporal activity data are continuous for both datasets. The detailed descriptions about the data attributes can be found in “Appendices A.6” and “A.7”. For the classification, we have converted the continuous data to discrete form by removing the timestamp feature. Furthermore, we have removed participants' data which are less than one month, redundant, noisy, incomplete, or missing. In the public Fitbit datasets, we have decided to consider activity data for 33 participants as they have performed activities for more than a month, resulting in 1397 records in total. In the MOX2-5 private datasets, we have considered data for 16 participants as they have performed activities more than a month, resulting in 539 records in total (see details in “Appendix A.6”).

#### Statistical testing

Normality test with methods, such as Shapiro–Wilk, D’Agostino’s Kˆ2, and Anderson–Darling test^44^ on each feature of the datasets have revealed that data samples do not have Gaussian characteristics. The normality test has been performed following the hypothesis testing method with *P* value > α = 0.05 (i.e., sample looks like gaussian) and *P* value < α = 0.05 (i.e., sample does not look like gaussian)^44^.

#### Feature selection for individual datasets

For the feature selection, we have performed methods, such as SelectKBest, recursive feature elimination (RFE), principal component analysis (PCA), ExtraTreesClassifier, ML pipeline with PCA and SelectKBest, and the correlation analysis. SelectKBest is a univariate feature selection and feature ranking method with statistical testing (e.g., chi-squared). RFE selects optimal features and assigns a rank after removing redundant features recursively. PCA is an unsupervised data reduction method that uses linear algebra to reduce data dimensions. It ranks features based on variance ratio. ExtraTreesClassifier is a bagging-based feature importance (or ranking) method.

Moreover, correlation analysis is a statistical method used to measure the strength of the linear relationship between two variables and compute their association. A high correlation signifies a strong relationship between the two variables, and a low correlation means that the variables are weakly related. The sample correlation coefficient (r) measures the closeness of association of the variables. "r" ranges from − 1 to + 1, where − 1 indicates a perfectly linear negative, i.e., inverse, correlation (sloping downward) and + 1 shows a linear positive correlation. "r" close to 0 suggests little, if any, correlation. Correlation methods are of the following two types: (a) Pearson correlation: it evaluates the linear relationship between two continuous variables, (b) Spearman correlation: It considers the monotonic or non-Gaussian relationship. Our used datasets have shown a non-Gaussian relationship with normality testing methods. The correlation analysis^45^ with the “spearman” method revealed the strength of the linear relationship between features and helped to determine which feature to retain or not^44,46–49^. We have considered removing features if they showed a powerful dependency score (r ≥ 0.72).

#### Combining features from datasets

**Figure 6:**
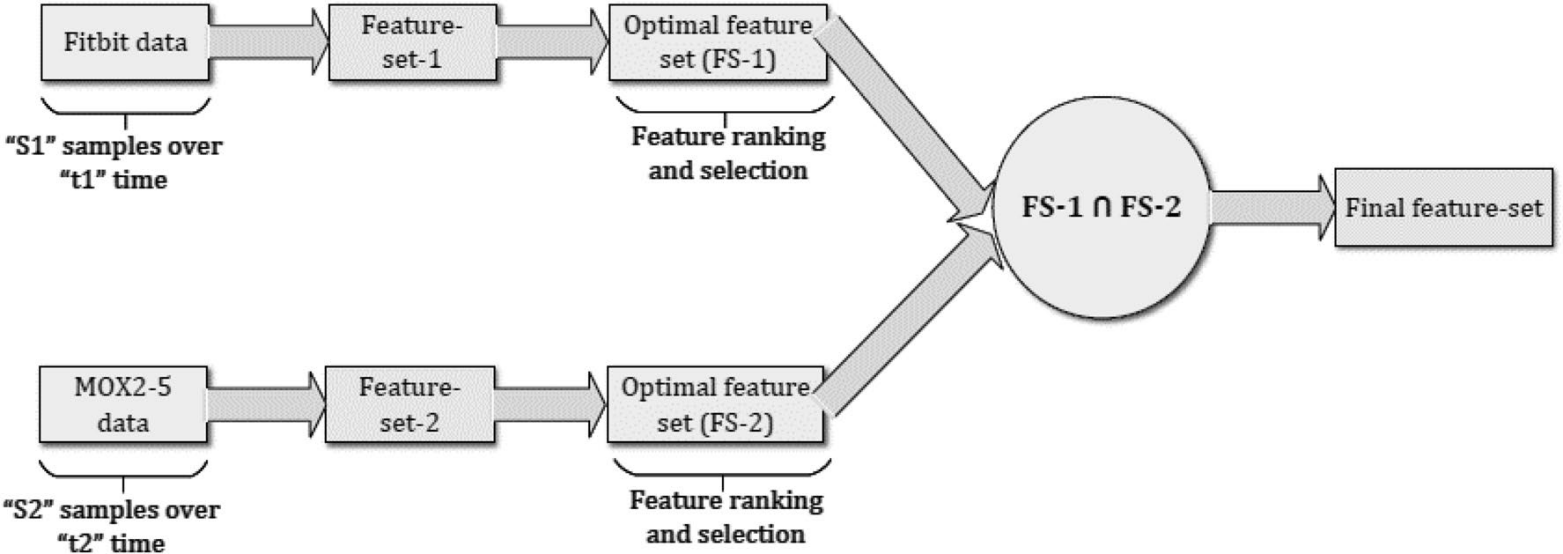
Combining features from both the datasets to prepare final feature-set.

First, we have performed feature ranking and feature selection from public Fitbit datasets based on adopted correlation method and created an optimal feature-set (FS-1). Second, we have performed the same feature selection method on private MOX2-5 datasets and created an optimal feature-set (FS-2). Then, we have performed an intersection of FS-1 and FS-2 to create a common feature space (or final feature-set) to make transfer learning approach relevant for this study (see Fig. 6).

#### Data labeling for classification

**Figure 7:**
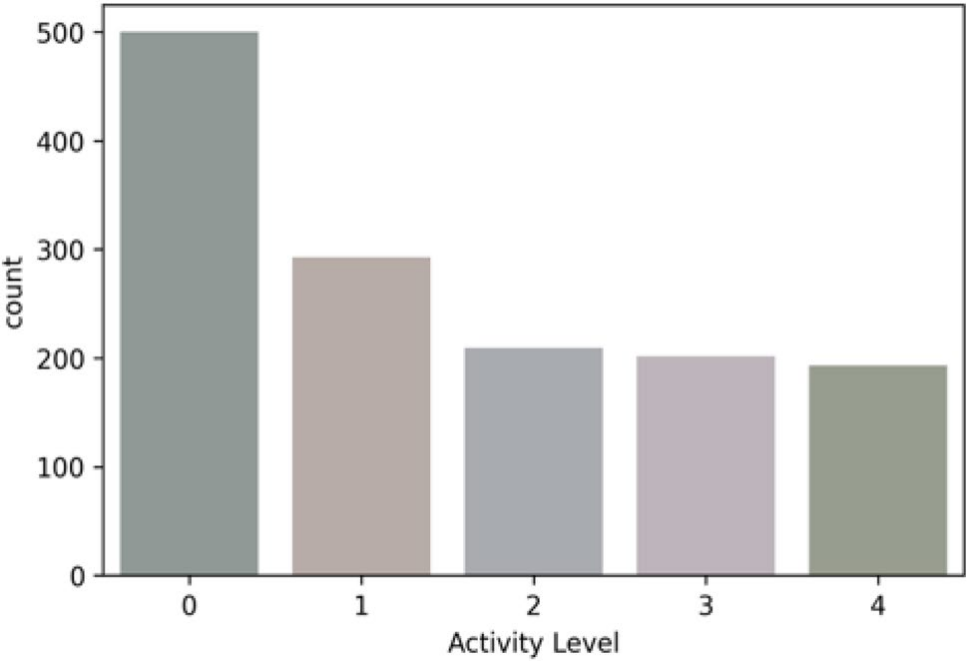
Class distribution for the public Fitbit datasets.

**Figure 8:**
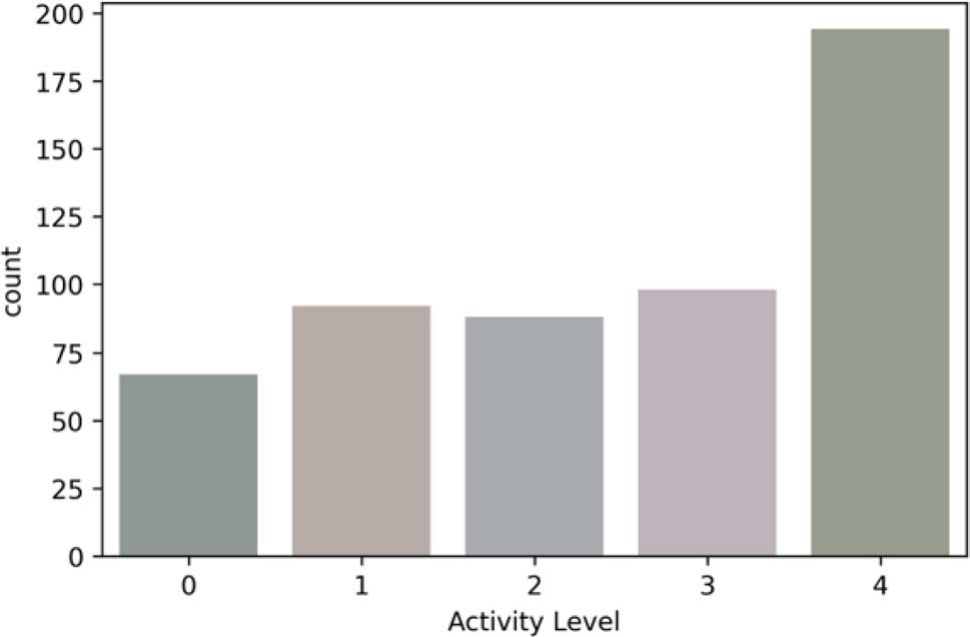
Class distribution for the private MOX2-5 datasets.

The “active” feature represents five classes—sedentary (0), low active (1), active (2), medium active (3), and highly active (4). The rule for the “Activity Level” feature class creation is defined in Table 2. We have created the feature class “Activity Level” based on the daily step-count following the standard guidelines^50–52^. In the multi-feature-based classification problem, we have derived the feature class “Activity Level” based on the steps, LPA, MPA, and VPA following the WHO activity guidelines for adults^5^. The features, such as age, gender, weight, weight-bearing, standing, are out of the scope. The class distributions in multi-class classification for both the datasets are depicted in Figs. 7 and 8.

### Model training and testing

#### ML models

This study has performed multi-class classification with standard ML algorithms, as in Table 6 and followed by an empirical comparison testing. We have used ML classifiers instead of deep learning classifiers because of the following reasons: convex optimization technique in gradient descent to find global minima, small amounts of training data, lesser model training time, training on a central processing unit (CPU), computationally inexpensive in terms of time and space, and transparency.

**Table 6:** Machine learning classifier models with optimization algorithms.

Models	Optimization algorithms
SVC (kernel = linear or rbf)	Gradient descent
Naïve Bayes (NB)	Gradient descent
Decision tree (DT)	Information Gain, Gini
K-nearest neighbor (KNN)	**‘**auto’, ‘ball_tree’, ‘kd_tree’, ‘brute’
Random forest (RF)	Ensemble—Bagging

Regularizations (L1-norm and L2-norm) have not been added to the models due to the limited set of features. *Support vector classifier (SVC)*^53^ is a supervised learning method to classify support vectors (or data points) using a decision plane or hyperplane to maximize the margin. It follows an iterative approach to generate the best hyperplane. SVC uses the “kernel” trick to convert low-dimensional input space to high-dimensional input space to make datapoints separable. The *Linear* kernel uses dot (.) product in two observation vectors. *Radial basis function (RBF)* kernel uses gamma (γ) (⋲ {0,1}) to map input datapoint space to infinite-dimensional space. *Naïve Bayes (NB)*^54^ is a supervised classification method based on the Bayes theorem, assuming that features are independent of each other. In Bayesian classification, posterior probabilities are determined to decide which feature-set will belong to which class based on the prior probabilities. NB can be classified into the following categories based on the nature of datasets: Gaussian, multinomial, and Bernoulli. Both SVC and NB are efficient classifiers on low data volume. *K-nearest neighbors (KNN)*^55^ is a supervised non-parametric method to cluster similar group data points based on different distance metrics, such as Euclidean, Hamming, Manhattan, and Minkowski. *Decision Tree (DT)*^54,55^ is a supervised predictive model to classify datasets based on the conditions. In classification DT, the decision variables at each node are categorical. It uses either “Gini” or “Entropy” criteria to generate binary splits. *Random forest (RF)*^55^ classifier uses a bagging ensemble learning method that combines multitude of decision trees during training time to improve prediction accuracy and reduce model variance. The final prediction of RF model is determined by estimating the average of all predictions from the individual estimators.

Furthermore, we have used a DummyClassifier as a simple baseline to compare against other more complex classifiers as mentioned in Table 6. It makes predictions that ignore the input features. We have used its strategy parameter as “most_frequent”.

### Training and testing

To better utilize the data, initially, we have shuffled the dataset, then split the dataset into training and testing. To boost the performance of the machine learning model, we have used a k-fold cross-validation where k ≥ 5. Furthermore, we have used Grid Search^56^ hyperparameter optimization technique for model tuning. The technique has helped in an appropriate selection of learning rate (alpha (α)) in the gradient descent algorithm, and a proper selection of other components, such as criterion, and max_depth in the tree-based models. Gradient descent follows a convex optimization technique with an upper limit (L) and a lower limit (µ) on curvature f:

2 $$\upmu {\text{I}}_{{\text{d}}} \le {\text{d}}^{2} {\text{f}}\left( {\text{x}} \right) \le {\text{LI}}_{{\text{d}}} ,$$

where d^2f.^(x) is the Hessian (H), µ > 0, I = Identity matrix, and L = Lipschitz continuous.

We have executed each ML classification model for five times and calculated their mean performance scores for a comparative analysis. The general pseudocode we have used to train and test the classifier models is stated as follows: 
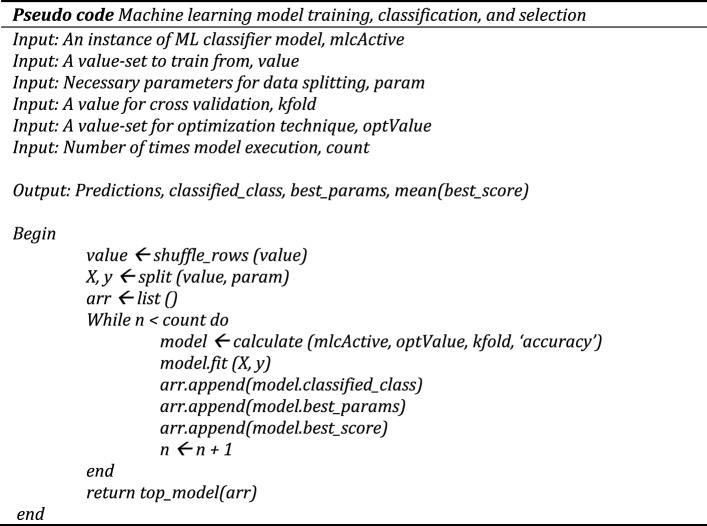


### Evaluation metrics

In this multi-class classification problem, we have focused only on discrimination analysis. The discrimination analysis metrics are precision, recall, specificity, accuracy score, F1 score, classification report, and confusion matrix. A confusion matrix is a 2-dimensional table (“actual” vs “predicted”), and both dimensions have “True Positives (TP)”, “False Positives (FP)”, “True Negatives (TN)”, and “False Negatives (FN)”^44,46–49^. Equations to calculate classification metrices are:

3 $${\text{Accuracy}}\left( {\text{A}} \right) = \frac{{\left( {{\text{TP}} + {\text{TN}}} \right) }}{{\left( {{\text{TP}} + {\text{FP}} + {\text{FN}} + {\text{TN}}} \right)}},\;0 \le \frac{{\left( {\text{A}} \right) }}{{\left( {100} \right)}} \le 1$$

4 $${\text{Precision}}\,\left( {\text{P}} \right) = \frac{{\left( {{\text{TP}}} \right){ }}}{{\left( {{\text{TP}} + {\text{FP}}} \right)}}$$

5 $${\text{Recall}}\,\left( {\text{R}} \right)\;{\text{or}}\;{\text{Sensitivity}}\;\left( {\text{S}} \right)\;{\text{or}}\;{\text{True}}\;{\text{positive}}\;{\text{rate}} = \frac{{\left( {{\text{TP}}} \right){ }}}{{\left( {{\text{TP}} + {\text{FN}}} \right)}}$$

6 $${\text{Specificity}}\,\left( {\text{S}} \right) = \left( {{1} - {\text{Sensitivity}}} \right) = \frac{{\left( {{\text{TN}}} \right){ }}}{{\left( {{\text{TN}} + {\text{FP}}} \right)}}$$

7 $${\text{F}}1\;{\text{score}}\,\left( {{\text{F}}1} \right) = \frac{{\left( {2*{\text{P}}*{\text{R}}} \right) }}{{\left( {{\text{P}} + {\text{R}}} \right)}},\quad 0 \le \frac{{\left( {{\text{F}}1} \right) }}{{\left( {100} \right)}} \le 1$$

8 $${\text{Matthew's}}\;{\text{correlation}}\;{\text{coefficient}}\left( {{\text{MCC}}} \right) = \frac{{\left( {{\text{TP}}\left( {{\text{TP}}*{\text{TN}} - {\text{FP}}*{\text{FN}}} \right) } \right) }}{{\surd \left( {\left( {{\text{TP}} + {\text{FP}}} \right)\left( {{\text{TP}} + {\text{FN}}} \right)\left( {{\text{TN}} + {\text{FP}}} \right)\left( {{\text{TN}} + {\text{FN}}} \right)} \right) }}, - 1 \le \frac{{\left( {{\text{MCC}}} \right) }}{{\left( {100} \right)}} \le + 1.$$

Accuracy tells how close a measured value is to the actual one. Recall or sensitivity suggests the exact number of positive measures. Precision means how relative the measured value is to the actual one. Furthermore, we used cross-validation score to determine overfitting and underfitting, validation curve to determine bias versus variance, and learning curve to visualize the convergence status of training score with the cross-validation score. Bias is an error due to erroneous assumptions in the learning algorithm, and variance is an error from sensitivity to small fluctuations in the training set. High bias leads to underfitting, and the high variance results in overfitting. Accuracy and F1 scores can be misleading because they do not fully account for the sizes of the four categories of the confusion matrix in the final score calculation. MCC is more informative than the F1 score and accuracy because it considers the balanced ratios of the four confusion matrix categories (for example, true positives, true negatives, false positives, and false negatives). The F1 score depends on which class is defined as a positive class. However, MCC does not depend on which class is the positive class, which has an advantage over the F1 score and avoids incorrectly defining the positive class^57^.

We have also tested if the standardization technique on the entire dataset before learning and the “Feature Union” tool during feature extraction can improve the performance of the models or not by reducing data leakage (if any!). In standardization scaling technique, values spread with mean (µ) = 0 and standard deviation (SD or σ) = 1. Furthermore, our ontology model has been evaluated against reasoning time, and SPARQL query execution time in Protégé and Jena Fuseki server. We have used reasoner, such as HermiT, Pellet, Fact++, RacerPro, and KAON2 available in Protégé for the verification of structural and logical consistencies in the ontology model.

### Rules for recommendation generation

A knowledge base is a database for knowledge management and provides means for information to be collected, organized, shared, searched, and inferred. It comprises two types of statements: asserted and inferred. The inferred statements are logical consequences of asserted statements and logical rules. A knowledge base is used to store and manipulate knowledge in computer science, interpreting invaluable information. It is often used in artificial intelligence applications and research for better understanding of a subject in computer-understandable form using appropriate symbols. In general, a ruleset is applied to the systems that involve human-designed or managed sets of rules. A knowledge base may contain semantic rules with human-understandable symbols and words. Semantic misunderstanding occurs when people assign different meanings to the same word or phrase. In this study, we have stored semantic rules (see “Appendix A.3”) in a knowledge base that combines ML classification results with preference variables to generate personalized recommendations based on the query execution in the SPARQL query engine (see Fig. 5).

### Conceptualization of automatic activity coaching

The eCoach prototype system aims to collect individual activity data from wearable activity sensors at a daily level (day-n) and classify the activity data into the identified five classes using ML algorithms. In the procedure, participants can set personal preferences in the eCoach mobile app for personalized recommendation generation and its meaningful delivery. In this study, we have focused on the conceptualization of weekly short-term activity recommendation generation in eCoaching.

Different classification models are available; however, we can’t determine “a priori” which classifier will perform the best. It requires enormous data for training, validation, and testing. We have collected real-activity data for sixteen adults using the MOX2-5 activity sensor over minimum of thirty days. According to the device manufacturer (Maastricht Instruments B.V.), the device's wear locations are thighs, hips, arms, and sacrum. We have placed the device in the trouser pocket for this study to collect activity data from the hip or thigh location. We use the MOX downloadable android mobile application to capture personal activity parameters into the download folder of the android smartphone or tablet. Then, we use our developed android supported eCoach mobile application to periodically transmit activity data to the eCoach back-end server. The eCoach server is protected by “eduVPN” and a firewall to filter redundant traffics. The back-end data collection module at the eCoach server has been developed with the Spring-boot framework (V. 2.5.x). The front-end (user interface) and the back end of the eCoach system is connected using REST (representational state transfer) microservice APIs (application programming interfaces). We have set up PostgreSQL (V. 12.9) for data storage at eCoach server following an authentication and authorization mechanism. The machine learning module for real-time model training, classification, model saving as pickle files, and model re-training has been developed with Python V 3.8.5. At the end of ML model execution, results for individual participants are stored in the database to display with android eCoach app. In the eCoach app, we have developed a push-notification generation mechanism with Google Material Design guidelines to notify participants about their daily or weekly, or monthly progress in activities and thereby, generate personalized recommendations to motivate them to reach short-term and long-term activity goals.

Sixteen participants used the MOX2-5 devices regularly to record their daily activity data during the study in progress. We have applied a mean imputation method to imputing missing activity data during real data collection. The format of the collected data from the MOX2-5 is comma-separated values (CSV). All the activity data have been captured “per minute” basis. The developed eCoach app reads those files periodically using a schedular service to send them in the data collection module for storage following a consistency check. The ML module has further processed individual data from “per minute” to “per hour” and stored it in a separate table. As a result, it has produced a set of 539 records, which is not sufficient to determine the factual accuracy of the best classifier model. Therefore, we have adopted the concept of transfer learning and incremental learning. Initially, we have trained all the potential classifiers (see Table 4) with public Fitbit datasets using Kfold = 5 cross-validator and radom_state = 7. Afterward, we have selected the best performing classifier and saved them as pickle files. Then, we have re-used those pre-trained models for individual activity level classification. In this study, we have classified the MOX2-5 sensor data with collection duration of more than 30-days with the following method—

- Pre-train model with public Fitbit datasets and select the best classifier,
- Training of pre-trained models with individual activity data (collected with MOX2-5 sensor) and model storing for individual participants, and
- Activity classification for day-n with individual models and re-train the models with the individual classification result of that day for the following day (e.g., day-n + 1) classification.

We have selected the activity classification result from the individual classifiers with the highest mean accuracy. The adopted transfer learning and incremental learning approaches have been expressed with the following pseudo code. 
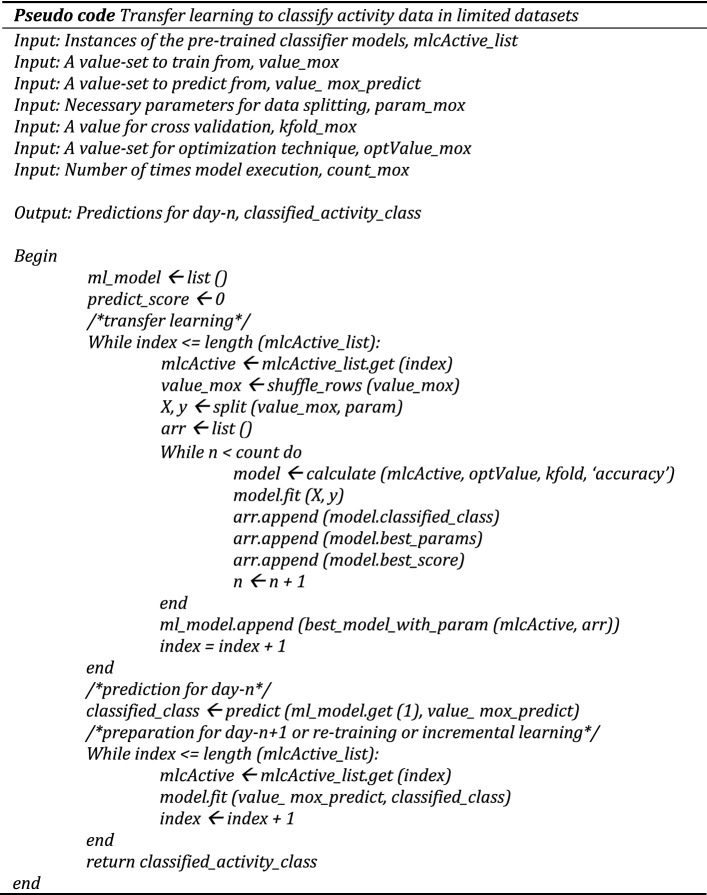


**Figure 9:**
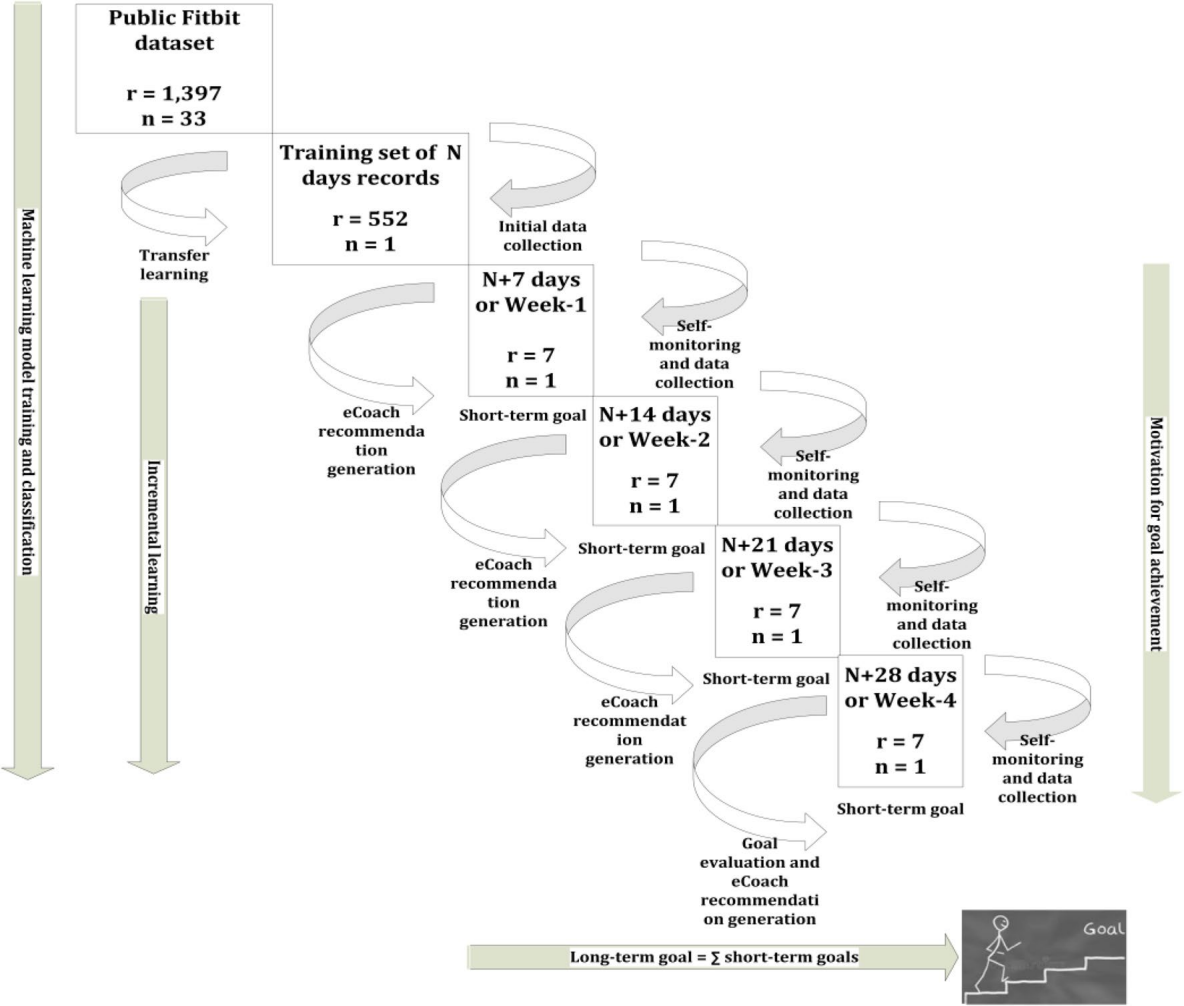
The usefulness of eCoaching to achieve short-term goals and long-term goal with transfer learning and incremental learning or training approaches.

The usefulness of the eCoaching concept to achieve personalized short-term goals (e.g., weekly) and long-term goal (e.g., 4 weeks) with transfer learning and incremental learning approaches have been conceptualized for an individual in Fig. 9, where “n” signifies the total number of individuals, “N” signifies total number of day’s data used for transfer learning, and “r” signifies the total number of daily activity record during model training and classification.

### Validation study

#### Verification of the classifiers

To verify the performance of the classifiers in public Fitbit and MOX2-5 datasets, we have the following three visualization approaches: (a) validation curve: it is an essential diagnostic tool that shows the sensitivity between changes in the accuracy of an ML model and changes in specific model parameters. A validation curve is usually drawn between some model parameters and the model's score. There are two curves in the validation curve—one for the training set score and one for the cross-validation score. Validation curves evaluate existing models based on hyperparameters, (b) learning curve: it shows the estimator's validation and training scores for different numbers of training samples. It is a tool to see how much we benefit from adding more training data and whether the estimator suffers more from variance or bias errors, and (c) scalability: it defines the ability of a classifier to adjust the classification results with an increasing number of training samples.

#### Verification of personalized recommendation generation and visualization

For personalized recommendation generation in the eCoach prototype system, we have maintained individual personal preferences to understand personal interests (e.g., see Table 4). Preferences data are stored in the knowledge base. Participants can view and update their preference data in the eCoach mobile app. The design and specification of the eCoach mobile app using a user-centered design approach are beyond the scope of this study. To determine the weekly score of personal goal achievement, we have summed up daily activity score, and the measure of daily activity score is mentioned in Table 2.

### Ethical approval and consent to participate

For our project, we received ethical approval from Norwegian Centre for Research Data (NSD) (#797208) and Regional Committees for Medical and Health Research Ethics (REK) (#53224). For this study, informed or signed consents have been obtained from all the participants. Here, we have not disclosed any identifiable data of the participants using text, numbers, or figures.

## Results

### Experimental setup

We used Python 3.8.5 supported language libraries, such as pandas (v. 1.1.3), NumPy (v. 1.21.2), SciPy (v. 1.5.2), Matplotlib (v. 3.3.2), Seaborn (v. 0.11.0), Plotly (v. 5.2.1), scikit-learn or sklearn (v. 0.23.2), and Graph Viz (v. 2.49.1) to process data and build the machine learning models. We set up the intended Python environment in Windows 10 Enterprise system using Anaconda Distribution Individual Edition and used the Spyder 4.1.5 IDE for the development, debugging, and data visualization. We have used Protégé 5.x open-source editor for ontology design and implementation. Moreover, we have used the Jena Framework to query ontology classes, predicates, subject, and objects and captured the corresponding execution time. Figures 1, 2, 3, 4, 5, 6 and 9 were created with Microsoft Visio Professional 2021 software.

### Experimental results

This section describes—*first*, feature correlation and selection, *second*, the analyses on public Fitbit and private MOX2-5 datasets with ML classifier models, *third*, the selection of the best model with their best parameters to train MOX2-5 activity data for personalized activity classification, *forth*, evaluation of model accuracy under data preparation and feature extraction pipeline, and *fifth*, the performance analysis of ontology reasoners in Protégé.

**Figure 10:**
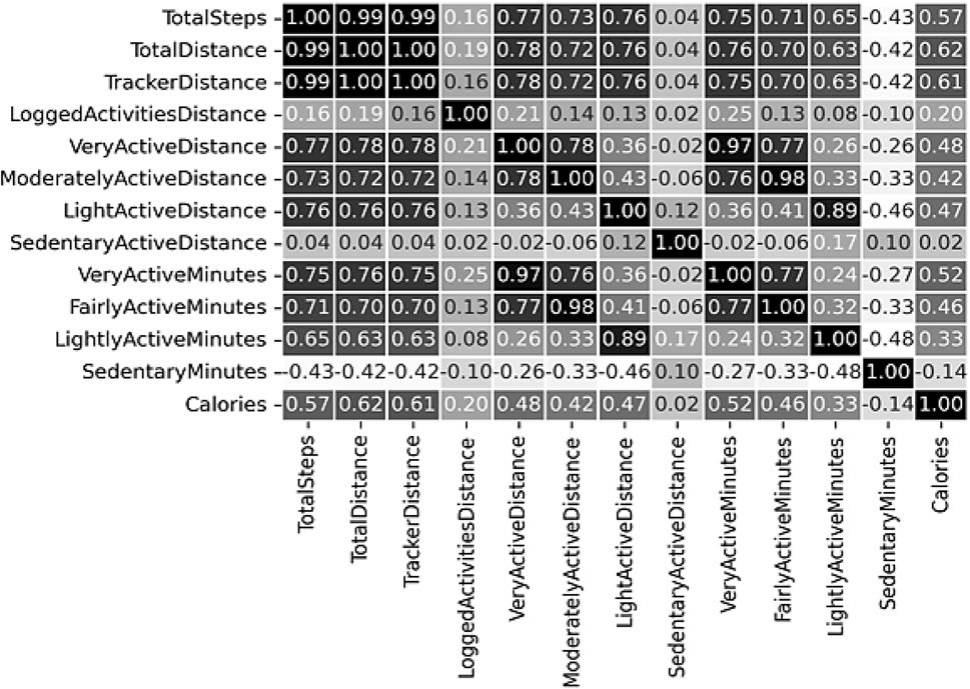
Correlation matrix for the public Fitbit datasets.

**Figure 11:**
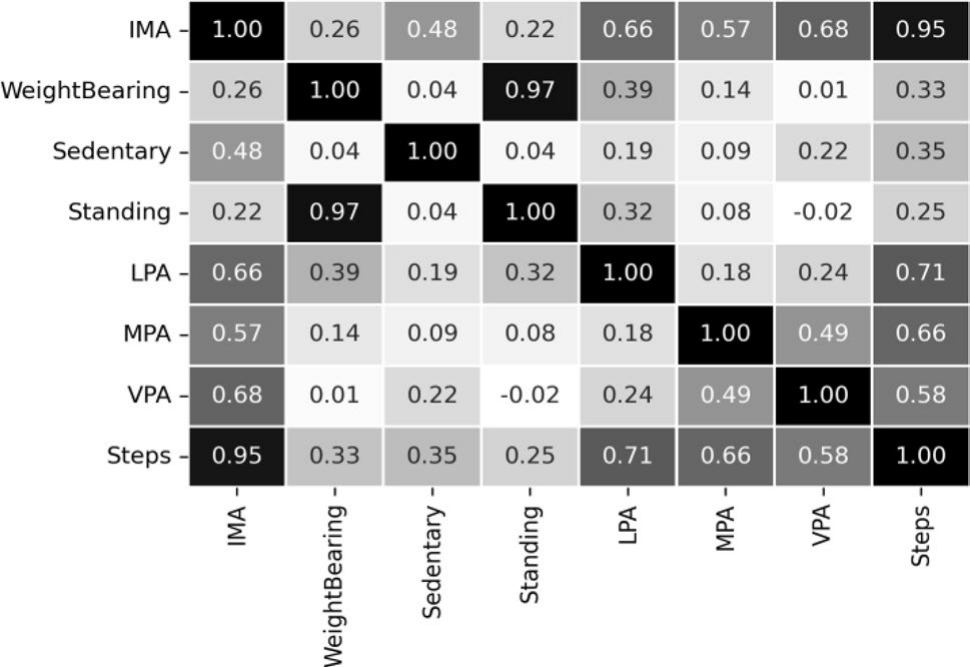
Correlation matrix for the private MOX2-5 datasets.

The correlation matrices obtained from both the datasets are depicted in Figs. 10 and 11 to show the strength of linear relationship between features to compute their association. We have prepared our final feature set (FFS) based on the outcome of feature selection methods and feature correlation score. The feature “calorie” is not related to the context of this study. Therefore, the final feature-set can be written as:

9 $${\text{FFS}} = \left( {{\text{FS - }}1 \cap {\text{FS - }}2} \right) = \left\{ {{\text{sedentary}},{\text{LPA}},{\text{MPA}},{\text{VPA}},{\text{steps}}} \right\}.$$

**Figure 12:**
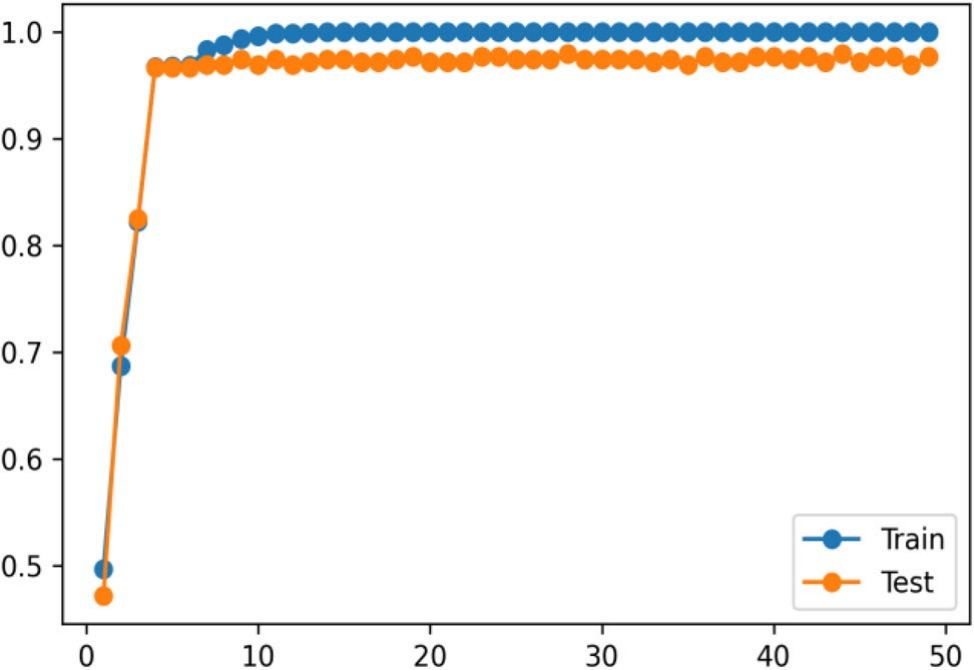
The training vs. testing curve for DecisionTreeClassifier in transfer learning.

**Figure 13:**
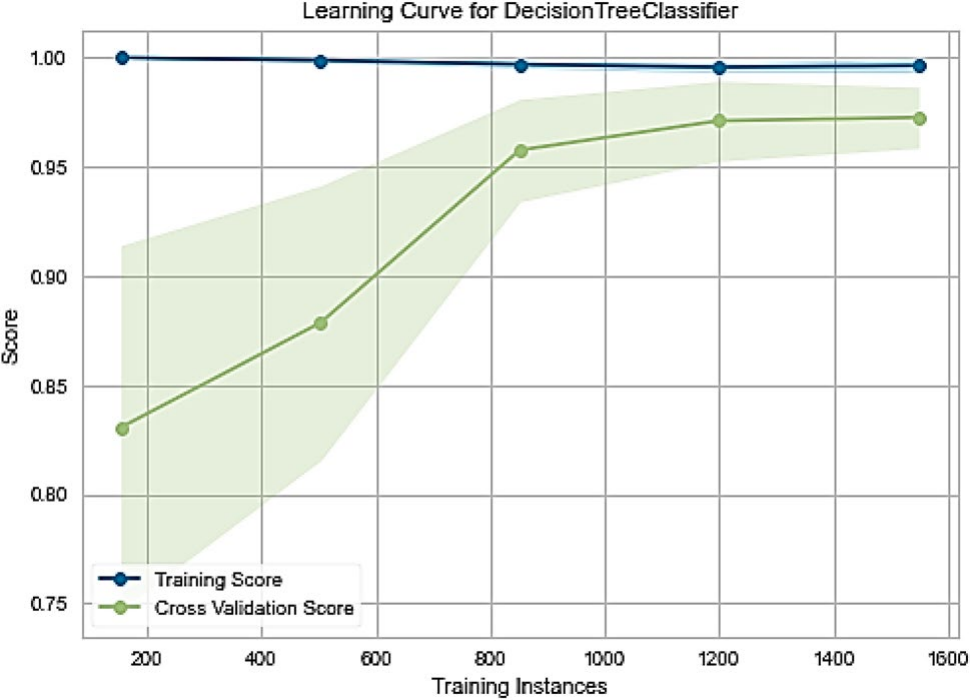
The learning curve for DecisionTreeClassifier in transfer learning.

**Figure 14:**
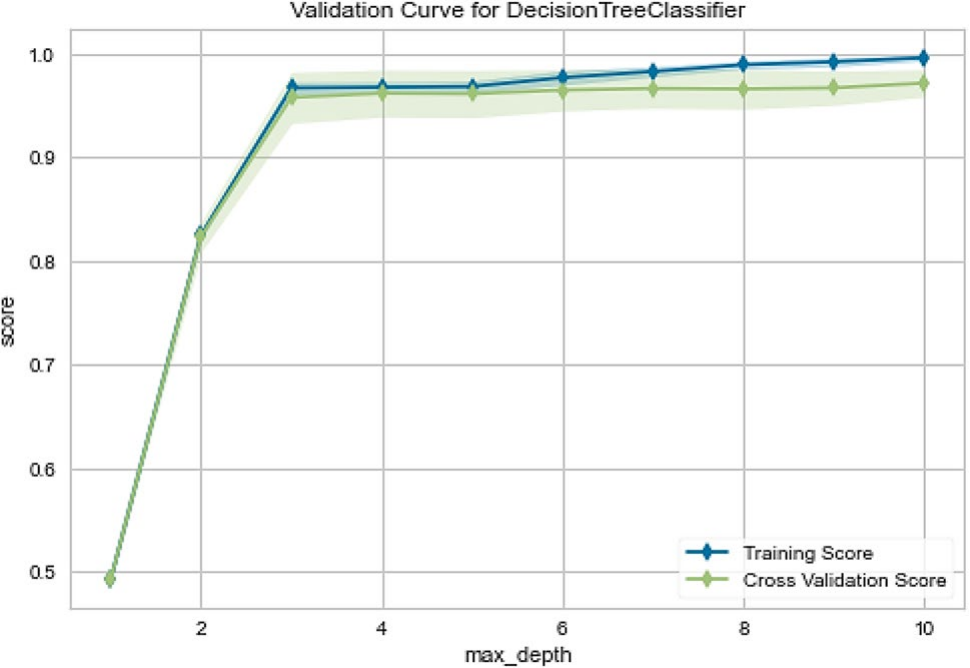
The validation curve for DecisionTreeClassifier in transfer learning.

**Figure 15:**
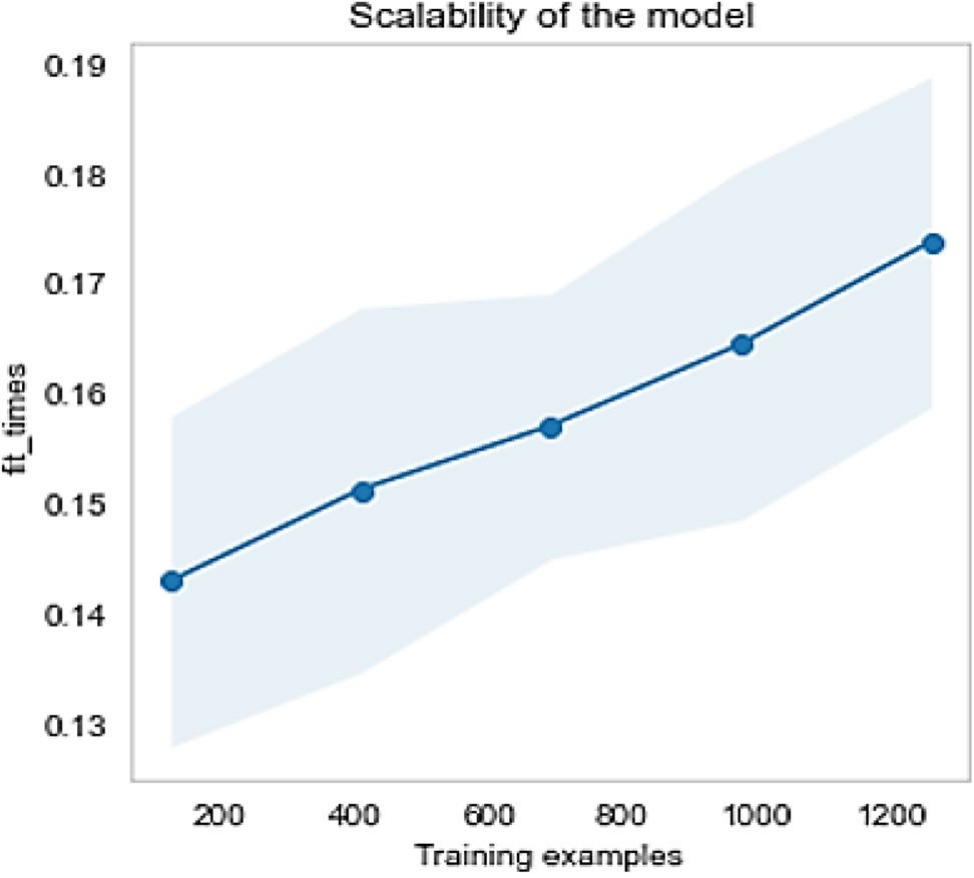
The scalability curve for DecisionTreeClassifier in transfer learning.

The Fitbit and MOX2-5 datasets were classified with all the classifiers mentioned in Table 6, and the results are presented in Tables 7 and 8. The best performing models are marked as bold. After that, we have used the best performing model in Fitbit datasets for transfer learning in MOX2-5 datasets, and the corresponding classification scores are in Table 9. The DecisionTreeClassifier with criterion “entropy” has outpaced other classifiers in all the datasets. Moreover, it has improved classification performance by ≈ 1.98% in transfer learning. The training vs. testing, validation, learning, and scalability curves for the DecisionTreeClassifier classifier used in transfer learning are depicted in Figs. 12, 13, 14 and 15. The results show neither overfit nor underfit and a sharp rise in model scalability with increasing training examples. Transfer learning has helped to improve ML model performance with trained knowledge transfer, saving resources and improving efficiency. The best hyperparameters (as obtained with the grid search method) for the DecisionTreeClassifier are described in Table 10 for each dataset.

**Table 7:** Performance of the machine learning classifiers for public Fitbit datasets.

ML classifier models	Mean accuracy	Precision	Recall	F1	MCC
SVC (kernel = 'linear')	90.00	90.00	90.00	90.00	87.16
SVC (kernel = 'rbf')	92.14	92.00	92.00	92.00	90.03
GaussianNB ()	81.78	82.00	82.00	82.00	76.82
DecisionTreeClassifier (criterion = "gini")	97.10	97.00	97.00	97.00	96.10
**DecisionTreeClassifier (criterion = "entropy")**	**97.50**	**97.00**	**98.00**	**97.00**	**96.78**
RandomForestClassifier (n_estimators = 50)	95.99	96.00	96.00	96.00	95.29
RandomForestClassifier (n_estimators = 100)	95.99	96.00	96.00	96.00	95.40
KNeighborsClassifier (n_neighbors = 2)	89.40	89.00	89.00	89.00	86.10
KNeighborsClassifier (n_neighbors = 4)	92.50	92.00	92.00	92.00	90.36
DummyClassifier	33.21	11.00	33.00	11.00	00.00

**Table 8:** Performance of the machine learning classifiers for private MOX2-5 datasets.

ML classifier models	Mean accuracy	Precision	Recall	F1	MCC
SVC (kernel = 'linear')	96.03	96.00	96.00	96.00	95.10
SVC (kernel = 'rbf')	47.01	43.00	47.00	45.00	30.69
GaussianNB ()	93.40	93.00	93.00	93.00	91.80
**DecisionTreeClassifier (criterion = "gini")**	**96.10**	**96.00**	**96.00**	**96.00**	**96.10**
**DecisionTreeClassifier (criterion = "entropy")**	**96.10**	**96.00**	**96.00**	**96.00**	**96.10**
RandomForestClassifier (n_estimators = 50)	95.80	95.00	96.00	95.00	94.88
RandomForestClassifier (n_estimators = 100)	95.80	95.00	96.00	95.00	94.88
KNeighborsClassifier (n_neighbors = 2)	70.98	70.00	71.00	70.00	66.31
KNeighborsClassifier (n_neighbors = 4)	66.54	66.00	67.00	66.00	61.30
DummyClassifier	35.18	35.00	35.00	35.00	00.00

**Table 9:** Performance of the machine learning classifiers after transfer learning.

ML classifier models	Mean accuracy	Precision	Recall	F1-score	MCC
SVC (kernel = 'linear')	84.70	85.00	84.00	84.00	80.29
SVC (kernel = 'rbf')	69.30	69.00	70.00	69.00	64.48
GaussianNB ()	64.40	64.00	65.00	64.00	57.29
DecisionTreeClassifier (criterion = "gini")	97.51	97.00	98.00	97.00	96.73
**DecisionTreeClassifier (criterion = "entropy")**	**97.99**	**98.00**	**98.00**	**98.00**	**96.79**
RandomForestClassifier (n_estimators = 50)	96.90	96.00	96.00	96.00	96.08
RandomForestClassifier (n_estimators = 100)	97.10	97.00	97.00	97.00	96.41
KNeighborsClassifier (n_neighbors = 2)	81.35	81.00	82.00	81.00	77.79
KNeighborsClassifier (n_neighbors = 4)	83.25	81.00	82.00	81.00	79.78
DummyClassifier	27.31	27.00	27.00	27.00	00.00

**Table 10:** Optimized parameters for top-5 models in single feature-based classification.

Data type	ML classifier models	Parameter list	Best parameters
Fitbit (public)	DecisionTreeClassifier (criterion = "entropy")	criterion = ['gini', 'entropy'] max_depth = [2,4,6,8,10,12]	criterion = ‘entropy, max_depth = 12
MOX2-5 (private)	DecisionTreeClassifier (criterion = "entropy")	criterion = ['gini', 'entropy'] max_depth = [2,4,6,8,10,12]	criterion = ‘entropy, max_depth = 4
Combined for Transfer learning	DecisionTreeClassifier (criterion = "entropy")	criterion = ['gini', 'entropy'] max_depth = [2,4,6,8,10,12]	criterion = ‘entropy, max_depth = 10

Furthermore, during training data preparation and feature extraction, we have investigated if the pipeline execution concept can improve the performance of the ML classifiers or not! Thus, we have created a data preparation pipeline models for the best performing classifier. We have normalized the whole datasets in each data preparation pipeline and then performed the classification. Similarly, we have created another feature extraction pipeline using the “FeatureUnion” that consisted of PCA and SelectKBest feature selection methods. Next, we have added the “FeatureUnion” and the classifier model in the pipeline to classify activity data. However, neither the data preparation pipeline nor the feature extraction pipeline significantly has improved the performance of the DecisionTreeClassifier in Fitbit datasets and combined datasets for transfer learning. However, it has improved the ML model performance in MOX2-5 datasets. The results of DecisionTreeClassifier in pipeline execution are in Table 11. Such pipeline approaches can be helpful for robust datasets, and further testing is required to prove the hypothesis in upcoming studies.

**Table 11:** Results of pipelined machine learning model execution.

Dataset type	Model	Accuracy score in data preparation pipeline	Accuracy score in feature extraction pipeline
Fitbit (public)	DecisionTreeClassifier (criterion = "entropy")	A = 96.06, P = 96.00, R = 96.00, F1 = 96.00, MCC = 95.30	A = 95.42, P = 95.00, R = 95.00, F1 = 95.00, MCC = 94.30
MOX2-5 (private)	DecisionTreeClassifier (criterion = "entropy")	A = 98.44, P = 98.00, R = 98.00, F1 = 98.00, MCC = 98.44	A = 98.44, P = 98.00, R = 98.00, F1 = 98.00, MCC = 98.44
Combined for Transfer learning	DecisionTreeClassifier (criterion = "entropy")	A = 97.21, P = 97.00, R = 97.00, F1 = 97.00, MCC = 97.21	A = 95.91, P = 95.00, R = 95.00, F1 = 95.00, MCC = 95.91

We have used OWL-full to read ontology in Jena in the “TTL” format and estimated the reading time to 1.0–1.5 s. Moreover, we have used TDB storage, “optimized rule-based reasoner OWL rules” and the Jena framework to query the ontology class, predicate, subject, and object of each sentence in < 1.2 s, < 1.8 s, < 1.7 s, and < 1.9 s, respectively. The estimated ontology reasoning time against different reasoners has been described in Table 12.

**Table 12:** Performance analysis of different reasoners available in Protégé.

Reasoner(s)	Average reasoning time (s)
HermiT	1.1–2.1
Pellet	2.2–4.1
Fact++	3.4–4.5
RacerPro	2.2–3.1
KAON2	2.9–3.8

## Discussion

### Principal findings

**Figure 16:**
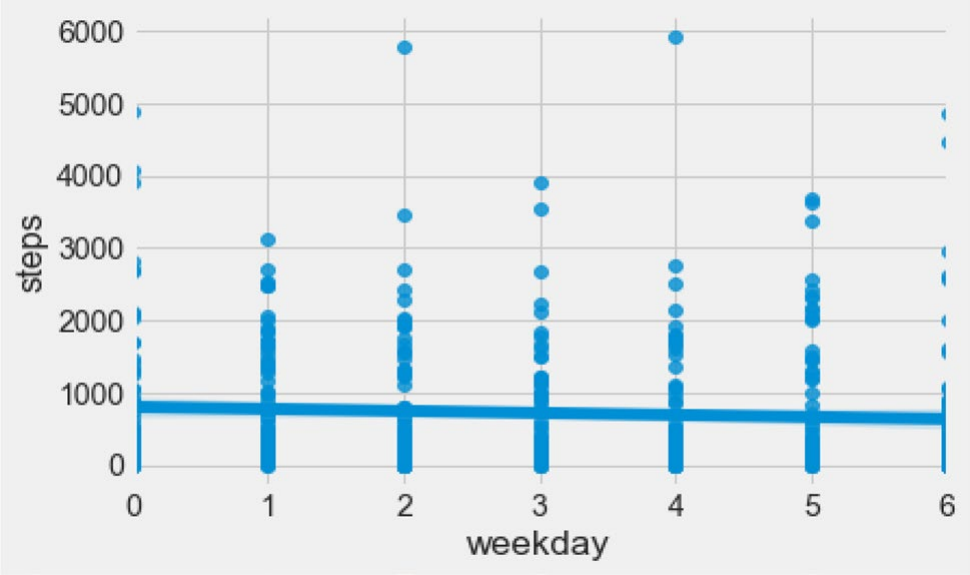
Weekly progression of activity in terms of daily step count.

Combined activity datasets have shown a trend of doing less exercise in terms of daily step count with the progression of a week, as shown in Fig. 16. A motivation with an eCoach may improve self-monitoring by keeping up an active pace of exercise over the days or weeks or months.

**Figure 17:**
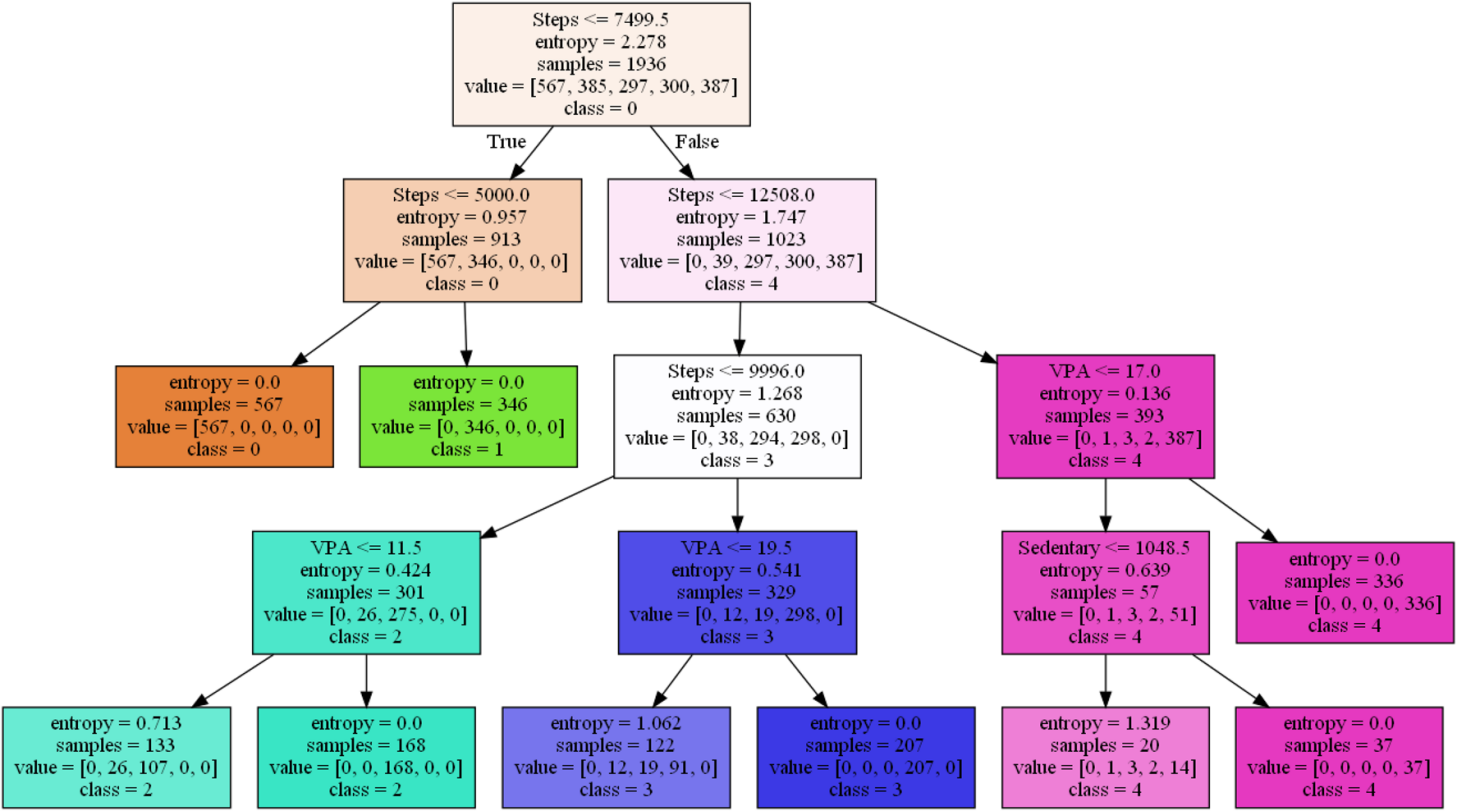
The interpretable classification visualization of DecisionTreeClassifier in transfer learning.

The performance of ML models depends on the nature of datasets used for a particular case study, under defined setting. Therefore, we have tried different ML classifiers with unique settings using the Grid-Search method; however, only DecisionTreeClassifier with criterion "entropy" produces the best accuracy, F1, precision, recall, and MCC score in our low-volume datasets for our case study. Entropy is an information theory metric that estimates the impurity or uncertainty in a group of observations. It decides how a decision tree chooses to split data. The most significant advantage of decision trees is that they make it very easy to interpret and visualize nonlinear patterns in data. They have worked faster than other classifiers in this exploratory data analysis. Moreover, decision trees do not require any data scaling or normalization. The interpretable classification visualization of DecisionTreeClassifier (with depth = 4) in transfer learning has been depicted in Fig. 17. The non-leaf nodes describe the reason for entropy-based branching; the leaf nodes are the predicted classes, and the branches hold binary values: True (1) or False (0). The entropy (E) in our system (S) can be expressed as:

10 $$E\left( S \right) = \mathop \sum \limits_{i = 0}^{c} - P_{i} \;log_{2} P_{i}$$

where “P_i_” is simply the frequentism of an element/class ‘i’ in our data.

Furthermore, this sub-section describes the overall process of daily and weekly score determination, goal verification, recommendation generation and its visualization on the eCoach app as a push notification. To verify the personalized recommendation generation in real participant data (i.e., MOX2-5 data), we have divided each participant’s activity days (n) into the following two parts—a. (n−7) days window for training the best performing classifier model(s) used in transfer learning, and b. remaining seven days window for testing with incremental learning approach. The incremental learning approach has helped in activity classification on the day-(n+1) based on model training with personal activity data up to day-n. We repeated the same incremental process until the goal periods were completed (here, we have assumed it as a window of 7-days). Moreover, we have used three standard emojis in recommendation visualization to motivate participants based on their weekly goal accomplishment [well done or good work (), up-to-the-mark or satisfactory performance () and improve performance (☹)].

**Figure 18:**
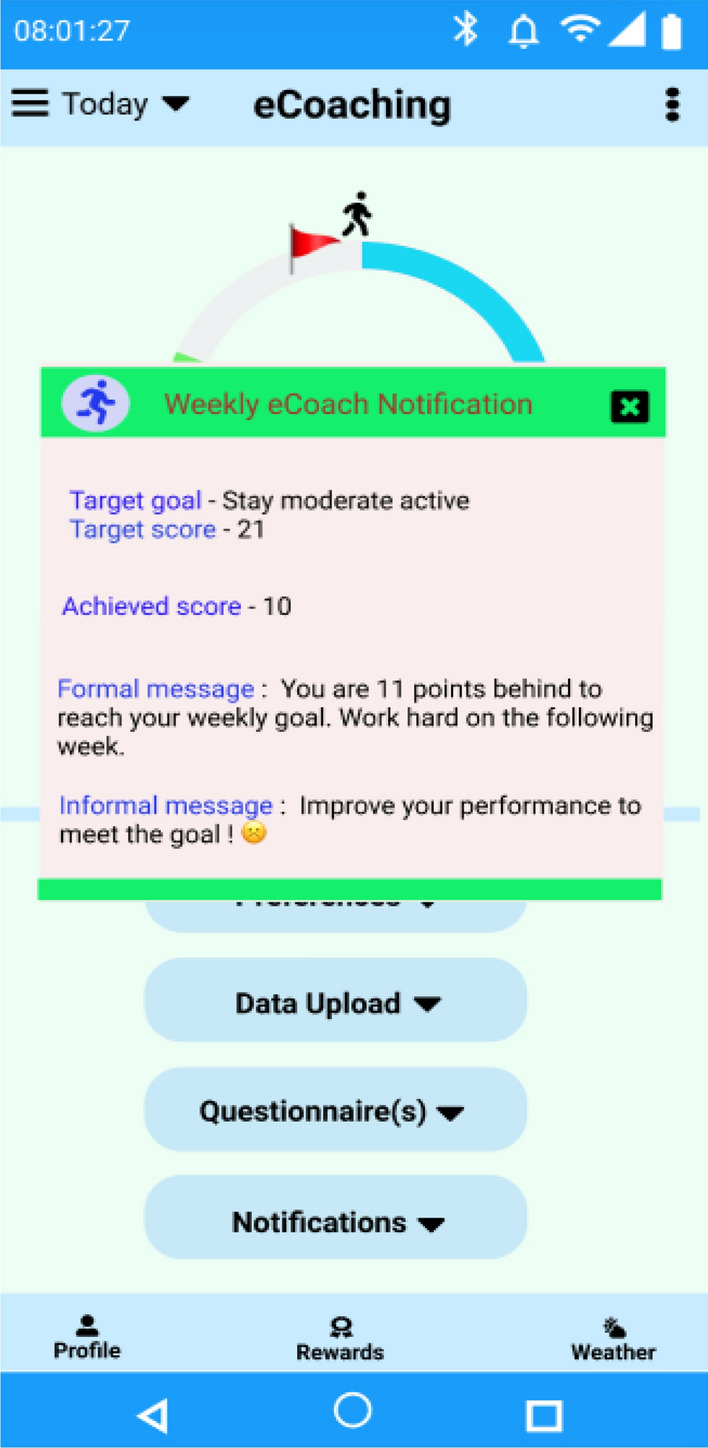
Weekly recommendation generation as a text (e.g., push notification) for P-1.

We have shown the overall process of recommendation generation and its meaningful presentation for a single participant (P-1) from the private MOX2-5 datasets (see Table 13, Fig. 18) collected for this study. The corresponding dataset for P-1 has been attached in “Appendix C” for verification. However, the same approach can be applied to other participants (P-2 to P-16). The transfer learning and incremental learning approach helped to increase the prediction accuracy on low-volume sensor datasets with limited feature space.

**Table 13:** Activity classification per day over a week duration for P-1 with transfer and incremental learning.

Days	The best performing classifier model(s) used in transfer learning	Actual activity level on day-n	Activity level predicted on day-n after incremental learning	Daily achieved score predicted	Propositional variable
Day-1	DecisionTreeClassifier (criterion = "entropy")	Low Active	Low Active	1	A-2, A-7, A-9
Day-2		Low Active	Low Active	1	A-2, A-7, A-9
Day-3		Medium Active	Medium Active	3	A-4, A-8
Day-4		Active	Active	2	A-3, A-9
Day-5		Low Active	Low Active	1	A-2, A-7, A-9
Day-6		Low Active	Low Active	1	A-2, A-7, A-9
Day-7		Low Active	Low Active	1	A-2, A-7, A-9
Weekly Score	Achieved = ∑Daily_achieved_score_predicted = (1 + 1 + 3 + 2 + 1 + 1 + 1) = 10				–
Prediction accuracy	∑Daily_achieved_score_actual—∑Daily_achieved_score_predicted = 0				–
Difference	Defined_goal_score—Achieved = (21 − 10) = 11				–
Weekly recommendation	***Formal message***—*“You are 11 points behind to reach your weekly goal. Work hard on the following week.*” ***Informal message***—*“Improve your performance to meet the goal!* ☹*”*				A-11

Recent coaching strategies are mainly based on the “String” messages that hardly personalize to each participant’s context, needs, and preferences. In this regard, our proposed ontology has been successful in modeling individualized recommendation message components, intent, and contents to label messages into different categories in an object-oriented design approach (see Fig. 3). The semantic rules described in “Appendix A.3” represent the logic behind recommendation message generation. The rule-based binary reasoning (If → 1, else → 0) helps to understand the formation of a personal activity recommendation messages on daily and weekly basis.

Overall, this study rigorously focuses on automizing the personalized activity recommendation generation with an ML pipeline, personal preference information, adjustable rule base, and their integration with a semantic network for reasoning and meaningful querying for personalized recommendations. Personalization is important in health recommendations to understand the user context and perspective. Therefore, health recommendations algorithms are contextually different from traditional user or item-based recommendation algorithms which are well accepted in the commercial domains. In Table 13, we have accomplished the efficiency of applying our proposed hybrid recommendation algorithm in activity eCoaching in an empirical way. The *Daily achieved score predicted* column in Table 13 describes the reason behind the recommendation generation in *Propositional variable* column. Such a study in eCoaching has not been conducted according to the existing literature. Therefore, we have restricted Table 1 to a qualitative comparison instead of an empirical comparison.

### Limitations and future scope

Our used datasets are small, and we thought they might be biased. High bias leads to model underfit. Therefore, we have used the MCC metric to understand the ML models' performance in a better way. However, more data is required to better train and test the classifier models. Multiple feature-based activity classification is more realistic than single feature-based classification (e.g., daily step count-based), as step count cannot be the only measure for activities. Activities like spot-running and MOX2-5 calculate appropriate IMA values; however, lower step counts than expected. In contrast, IMA appropriately correlates with step count in walking or running. However, this study has elaborated activity-level classification strategies, semantic knowledge representation, tuple query processing, and meaningful, personalized recommendation generation. However, this is not actual coaching but conceptual modeling with machine learning algorithms and semantics. In authentic coaching, to attain a weekly or monthly goal, as a part of continuous monitoring, the eCoach module will generate personalized recommendations on time, based on the activity outcome on each day, followed by a predictive analysis to achieve the weekly goal. For evaluating the practical effectiveness of the concept, a further study is required on a cluster of controlled trials.

Collaborative filtering^58–60^ is a popular recommendation generation technique to filter out items based on the reactions of similar users. Collaborative filtering is a searching problem where a large group of users is being searched to find a smaller set of users with tastes like a particular user. It helps to create a ranked recommendation. This paper proposes a model-based personalized recommendation algorithm based on a hybrid approach where the ML classification results are combined with semantic ontology to generate rule-based customized recommendations. Activity recommendations are filtered out based on personal preferences and goal achievements. The ontology tree structure explains the logic or rule behind a particular recommendation generation. The process is very personalized and, therefore, does not include the concept of group similarity in recommendation generation. In future, we will extend this study with a group-based metaheuristic approach by combining the idea of collaborative filtering. We will analyze further the applicability of density-based spatial clustering, session, criteria, and statistical models^61–63^ in our future group-based lifestyle recommendation generation.

## Conclusion

This study has shown a direction to use ML technology, personal preferences, and semantic ontology to design and develop an intelligent eCoach system with semantic knowledge representation to generate automatic, meaningful, contextual, and personalized activity recommendations to attain personal activity goals. According to this theoretical research, the improvement of physical activity in sequence with wearable activity sensors, digital activity trackers, and eCoach features can be encouraging. The concept, such as transfer learning, exists in image processing; its re-use with incremental training and testing in an activity eCoaching idea has been effective. Ontology has increased the logical consistency of our eCoach model with an object-oriented design. This study has presented a detailed analysis of different ML classifiers on activity data, thereby generating understandable and meaningful personalized activity recommendation generation with sematic rules and SPARQL query execution. We will extend this study with the integration of concepts such as step prediction, statistical methods, activity density, clustering, and probabilistic interval predictions to make eCoach recommendations more realistic and evidence based.

## Supplementary Information

Supplementary Information 1.

Supplementary Information 2.

Supplementary Information 3.

## Data Availability

All the data used or produced in this study are either in the main text or in the supplementary files. All the codebase and datasets will be made publicly available, and the corresponding author AC can be contacted for the datasets. As a sample, we have made MOX2-5 dataset public for Participant-1 (P-1) available in “Supplementary Information 3”. Further data will be made available in the extended version of this paper.

## Abbreviations

eCoach: Electronic coaching
ICTs: Information and Communication Technologies
ML: Machine learning
LPA: Low physical activity
MPA: Medium physical activity
VPA: Vigorous physical activity
IMA: Activity intensity
SVC: Support vector classifier
KNN: K-nearest neighbor
RF: Random forest
WHO: World Health Organization
OWL: Semantic Web language
RDF: Resources description framework
SPARQL: SPARQL Protocol and RDF Query Language
OWA: Open-world assumption
NSD: Norwegian Centre for Research Data
REK: Regional Committees for Medical and Health Research Ethics
FFS: Final feature set
MCC: Matthew’s correlation coefficient
