# Author Correction: Machine learning and ontology in eCoaching for personalized activity level monitoring and recommendation generation

**DOI:** 10.1038/s41598-023-30029-9

**Published:** 2023-02-20

**Authors:** Ayan Chatterjee, Nibedita Pahari, Andreas Prinz, Michael Riegler

**Affiliations:** 1 Department of Information and Communication Technology, Centre for e-Health, University of Agder, Jon Lilletuns Vei 9, Grimstad, Norway; 2 Department of Holistic Systems, Simula Metropolitan Center for Digital Engineering (SimulaMet), Oslo, Norway; 3 Department of Software Engineering, Tietoevry Norway AS, Oslo, Norway

Correction to: *Scientific Reports* https://doi.org/10.1038/s41598-022-24118-4, published online 18 November 2022

The original article of this Article contained a repeated error where “private MOX2-5 dataset” was incorrectly given as “real-time private MOX2-5 dataset” throughout the article.

Also, the Data availability section was incomplete.

“All the data used or produced in this study are either in the main text or in the supplementary files. All the codebase and datasets will be made publicly available, and the corresponding author AC can be contacted for the datasets.”

now reads:

“All the data used or produced in this study are either in the main text or in the supplementary files. All the codebase and datasets will be made publicly available, and the corresponding author AC can be contacted for the datasets. As a sample, we have made MOX2-5 dataset public for Participant-1 (P-1) available in “Supplementary Information 3”. Further data will be made available in the extended version of this paper.

The original Article has been corrected.

