# Supplementary material for: Machine learning and ontology in eCoaching for personalized activity level monitoring and recommendation generation
Source: Sci Rep. 2022 Nov 18;12:19825. doi: 10.1038/s41598-022-24118-4 (PMC9674665; doi:10.1038/s41598-022-24118-4)
Supplement: Supplementary file 1 — Supplementary Information 1. [file 41598_2022_24118_MOESM1_ESM.docx]

## Appendix A

**Table A.1**. A high-level description on the selected parameters.

| Parameter(s) | Descriptions |
| --- | --- |
| Hybrid recommendations | A hybrid recommendation system integrates multiple recommendation algorithms as a single unit. Solely data-driven or rule-based recommendation systems have several disadvantages in healthcare. In this study, we have adopted a hybrid recommendation architecture where data-driven prediction results are combined with rule-base. |
| Transfer learning | It is a research method in ML where stored knowledge is gained to apply in another model in solving a related problem. It is popular in image classification using Convolutional Neural Network (CNN); however, in this study, we have used it on the limited volume of activity dataset for activity level classification. |
| Incremental learning | It is a method used in ML pipeline for real-time processing on data where incoming data continuously grow to extend the knowledge of the existing model with further training. |
| Observation with activity sensor | Activity trackers record individual movements throughout the day. These sensors measure many parameters such as acceleration (e.g., steps taken during the day, distance traveled, duration of the activity, whether you are reaching your daily goals), frequency, duration, intensity, and patterns of individual movements. Then they are passed through Algorithms to do some guesswork to make sense of all the readings the data provides. Popular activity trackers are MOX2-5, Fitbit, Actigraph, Xiaomi Mi Band, Huawei Watch, Apple Watch, Samsung watch, AS Series (97, 98, 99). Wearable activity trackers are connected to the mobile app via Bluetooth short-range data transfer medium. |
| Preference settings | Preferences can be system-defined or user-defined (or generic). Preferences can be three types: goal setting, response type for intuitive coaching, and interaction type. Preferences settings are adequate for customizing tailored recommendation generation. Preference settings define nature of goals, type of response (e.g., direct vs. motivational, generic vs. personalized), mode (e.g., style, graph), frequency (e.g., hourly, quarterly, twice a day, once a day), and medium (e.g., audio, video, voice, text) of the interaction with an eCoach. |

**Table A.2.** Propositional variable and corresponding recommendation messages**.**

| Type | Propositional variable | Description |
| --- | --- | --- |
| A-1 | Sedentary | Please continue light activity (e.g., sports 1-3 days/week, a walking goal of 5,000 to 7,499 steps/ day) |
| A-2 | Low_physically_active | Please continue more activity (e.g., sports 3-5 days/week, a walking goal of 7,500 to 9,999 steps/ day) OR do at least 150-300 minutes (2.5 – 5 hours) of moderate-intensity aerobic exercise; or at least 75-150 minutes of high-intensity aerobic exercise or perform an equivalent combination of moderate and high-intensity activities within a week to stay active |
| A-3 | Physically_active | Please continue same or more activity based on your goal (e.g., sports 3-5 days/week, a walking goal of 7,500 to 9,000 steps/ day) |
| A-4 | Moderate_physically_active | Please continue same or more activity based on your goal (e.g., sports 3-5 days/week, a walking goal of 10,000 to 12,499 steps/ day) |
| A-5 | Vigorous_physically_active | Please continue same or more activity based on your goal (e.g., sports 5+ days/week, a walking goal of 12,500+ steps/ day) |
| A-6 | Sedentary_hour_negative | Please be active for z hr. more as today you were z hr. more sedentary beyond your goal. |
| A-7 | Sedentary_hour_positive | You were very active today and z hr. less sedentary; therefore, you can take that hr. of rest tomorrow. |
| A-8 | Daily_Goal_achieved | Good work. Please keep it up tomorrow. You are active and completed the goal for today.  Overview:  You have performed x steps today.  You were sedentary for z hrs.  You were m minutes of active. |
| A-9 | Daily_Goal_not_achieved | You must improve to meet the daily goal. Please stay active tomorrow.  Overview:  You have performed x steps today.  You were sedentary for z hrs.  You were m minutes of active. |
| A-10 | Weekly_Score_achieved | Good work. You are active and completed the goal for this week.  You have achieved adequate points to reach your weekly goal, or You are X points ahead of your weekly goal. Please keep it up next week. |
| A-11 | Weekly_Score_not_achieved | Improve your performance to meet the goal.  You are X points behind to reach your weekly goal. Work hard on the following week. |

**Table A.3.** In context recommendation conditions, and corresponding rules for test set-up.

| No. | Semantic Rule(s) [If] and Condition [Implies] |
| --- | --- |
| 1 | (hasPhysicalActivityLevel == 0) IMPLIES (Sedentary AND hasPhysicalActivityLevel)  (hasPhysicalActivityLevel == 1) IMPLIES (Low_physically_active AND hasPhysicalActivityLevel)  (hasPhysicalActivityLevel == 2) IMPLIES (Physically_active AND hasPhysicalActivityLevel)  (hasPhysicalActivityLevel == 3) IMPLIES (Moderate_physically_active AND hasPhysicalActivityLevel)  (hasPhysicalActivityLevel == 4) IMPLIES (Vigorous_physically_active AND hasPhysicalActivityLevel) |
| 2 | ((hasSedentaryBouts – daily_sedentary_goal_time as set in goal) > 0) IMLPIES (Sedentary_hour_negative)  ((hasSedentaryBouts – daily_sedentary_goal_time as set in goal) <= 0) IMLPIES (Sedentary_hour_positive) |
| 3 | ((hasMPAMinutes – daily_MPA_goal as set in goal) OR (hasVPAMinutes*2 – daily_VPA_goal as set in goal) => 0) IMLPIES (Activity_minute_positive)  ((hasMPAMinutes – daily_MPA_goal as set in goal) OR (hasVPAMinutes*2 – daily_VPA_goal as set in goal) < 0) IMLPIES (Activity_minute_negative) |
| 4 | ((hasMPAMinutes – daily_MPA_goal as set in goal) OR (hasVPAMinutes*2 – daily_VPA_goal as set in goal) => 0) AND ((hasSedentaryBouts – daily_sedentary_goal_time as set in goal) <= 0) IMLPIES (Daily_Goal_achieved) |
| 5 | ((hasMPAMinutes – weekly_MPA_goal as set in goal) OR (hasVPAMinutes*2 – weekly_VPA_goal as set in goal) => 0) AND ((hasSedentaryBouts – weekly_sedentary_goal_time as set in goal) <= 0) IMLPIES (Weekly_Score_achieved) |
| 6 | (Sedentary + Low_physically_active + Moderate_physically_active + Vigorous_physically_active + Sedentary_hour_negative + Sedentary_hour_positive + Daily_Goal_achieved + Daily_Goal_not_achieved + Weekly_Score_achieved + Weekly_Score_not_achieved + Good_weather + Bad_weather = 1) |

**Table A.4.** Attributes of the Zenodo Fitbit dataset.

| No | Attributes | Data type |
| --- | --- | --- |
| 1 | Id | Long |
| 2 | ActivityDate | String |
| 3 | TotalSteps | Integer |
| 4 | TotalDistance | Double |
| 5 | TrackerDistance | Double |
| 6 | LoggedActivitiesDistance | Double |
| 7 | VeryActiveDistance | Double |
| 8 | ModeratelyActiveDistance | Double |
| 9 | LightActiveDistance | Double |
| 10 | SedentaryActiveDistance | Double |
| 11 | VeryActiveMinutes (VPA) | Integer |
| 12 | FairlyActiveMinutes (MPA) | Integer |
| 13 | LightlyActiveMinutes (LPA) | Integer |
| 14 | SedentaryMinutes | Integer |
| 15 | Calories | Integer |

**Table A.5.** Attributes of the MOX2-5 dataset.

| No | Attributes | Type |
| --- | --- | --- |
| 1 | Date | String |
| 2 | Time | String |
| 3 | UploadStatus | Character |
| 4 | IMA | Integer |
| 5 | WeightBearing | Integer |
| 6 | Sedentary | Integer |
| 7 | Standing | Integer |
| 8 | LPA | Integer |
| 9 | MPA | Integer |
| 10 | VPA | Integer |
| 11 | Steps | Integer |

**Table A.6.** Private MOX2-5 activity data collection details for participants (n=16).

| Participant(s) | Duration of data collection (days) | Considered Total records | Total sedentary seconds | Total VPA seconds | Total MPA seconds | Total LPA seconds | Total steps |
| --- | --- | --- | --- | --- | --- | --- | --- |
| P1 | 43 | 43 | 3512792 | 8510 | 52196 | 183702 | 392512 |
| P2 | 48 | 48 | 4261190 | 50214 | 95730 | 200524 | 588132 |
| P3 | 30 | 30 | 2293208 | 24248 | 62502 | 65494 | 273708 |
| P4 | 31 | 31 | 3065884 | 15156 | 23402 | 254332 | 442365 |
| P5 | 30 | 30 | 2402790 | 43104 | 57606 | 123170 | 398029 |
| P6 | 30 | 30 | 2316338 | 51094 | 64885 | 77141 | 305673 |
| P7 | 39 | 39 | 3784340 | 78908 | 53876 | 245160 | 398296 |
| P8 | 31 | 31 | 3028756 | 112 | 38230 | 103480 | 252551 |
| P9 | 32 | 32 | 2623966 | 30722 | 72308 | 153174 | 419063 |
| P10 | 31 | 31 | 2395160 | 27024 | 58846 | 120820 | 347144 |
| P11 | 33 | 33 | 3061236 | 15432 | 45440 | 247896 | 436404 |
| P12 | 31 | 31 | 590028 | 25142 | 37680 | 151150 | 271888 |
| P13 | 31 | 31 | 2297915 | 10006 | 27487 | 135314 | 269258 |
| P14 | 30 | 30 | 1963218 | 14891 | 39670 | 193226 | 320134 |
| P15 | 38 | 38 | 925614 | 256896 | 58212 | 32272 | 411033 |
| P16 | 31 | 31 | 664302 | 18746 | 63638 | 187498 | 341063 |

**Table A.7.** Participant characteristics (n=16).

| Factors | Mean (µ) | SD (σ) | Min | Max |
| --- | --- | --- | --- | --- |
| Age | 35.375 | 7.03 | 21 | 51 |
| Height (cm) | 173.5 | 8.02 | 158.5 | 184.0 |
| Weight (Kg.) | 77.0 | 16.36 | 55.0 | 107.0 |
| BMI | 25.38 | 3.93 | 19.41 | 31.604 |
| Duration | 33.6875 | 5.41 | 30 | 48 |
| Total sedentary minutes | 2449171 | 1051610.5 | 590028 | 4261190 |
| Total VPA minutes | 41887.81 | 60688.5 | 112 | 256896 |
| Total MPA minutes | 53231.75 | 17965 | 23402 | 95730 |
| Total LPA minutes | 154647.1 | 66540.6 | 32272 | 254332 |
| Total steps | 366703.3 | 87202.25 | 252551 | 588132 |
